# Enzyme-Responsive Nanoparticles for the Targeted Delivery of an MMP Inhibitor to Acute Myocardial Infarction

**DOI:** 10.1021/acs.biomac.3c00421

**Published:** 2023-09-11

**Authors:** Holly L. Sullivan, Yifei Liang, Kendra Worthington, Colin Luo, Nathan C. Gianneschi, Karen L. Christman

**Affiliations:** † Shu Chien-Gene Lay Department of Bioengineering and the Sanford Consortium for Regenerative Medicine, University of California San Diego, La Jolla, California 92093, United States; ‡ Department of Chemistry, International Institute for Nanotechnology, Simpson-Querrey Institute, Chemistry of Life Processes Institute, Northwestern University, Evanston, Illinois 60208, United States; § Departments of Materials Science & Engineering, Biomedical Engineering and Pharmacology, Northwestern University, Evanston, Illinois 60208, United States; ∥ Department of Chemistry & Biochemistry, University of California San Diego, La Jolla, California 92093, United States

## Abstract

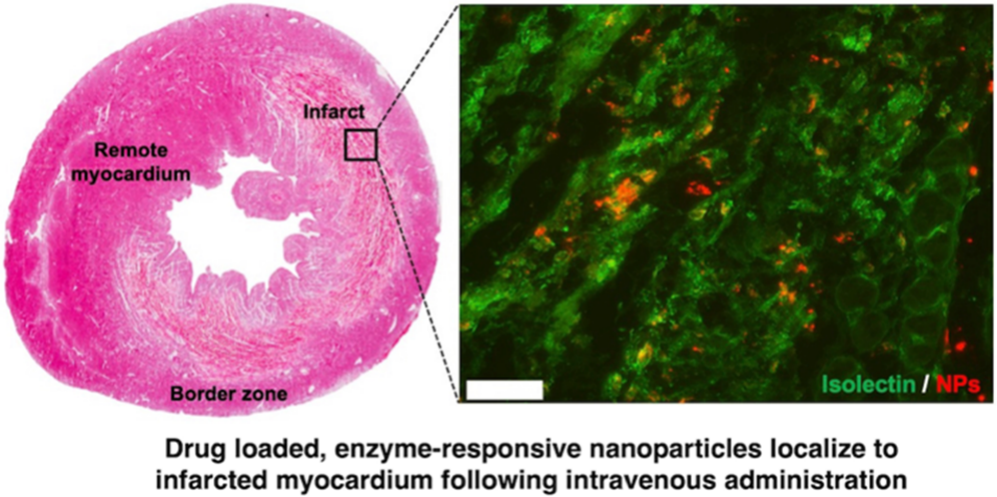

Herein, we have developed a drug-loaded matrix metalloproteinase (MMP)-responsive micellar nanoparticle (NP) intended for minimally invasive intravenous injection during the acute phase of myocardial infarction (MI) and prolonged retention in the heart for small-molecule drug delivery. Peptide-polymer amphiphiles (PPAs) bearing a small-molecule MMP inhibitor (MMPi), PD166793, were synthesized via ring-opening metathesis polymerization (ROMP) and formulated into spherical micelles by transitioning to aqueous solution. The resulting micellar NPs underwent MMP-induced aggregation, demonstrating enzyme responsiveness. Using a rat MI model, we observed that these NPs were capable of successfully extravasating into the infarcted region of the heart where they were retained due to the active, enzyme-mediated targeting, remaining detectable after 1 week post administration without increasing macrophage recruitment. Furthermore, *in vitro* studies show that these NPs demonstrated successful drug release following MMP treatment and maintained drug bioactivity as evidenced by comparable MMP inhibition to free MMPi. This work establishes a targeted NP platform for delivering small-molecule therapeutics to the heart after MI, opening possibilities for myocardial infarction treatment.

## 1 Introduction

Myocardial infarction (MI) leads to ischemic damage and cardiomyocyte death and is a leading cause of death globally.^1^ During acute ischemic injury, there is enhanced vascular permeability in the infarct.^2^ An upregulation of inflammatory enzymes including matrix metalloproteinases (MMPs) also leads to the degradation of the native extracellular matrix (ECM) within the heart, compromising mechanical support in addition to cardiomyocyte structure and function.^3^ MMP activity is upregulated within hours post-MI and can remain at high levels for weeks to months afterward.^4^ Despite the current standard of care, over time, ischemic tissue damage leads to negative left ventricle (LV) remodeling,^5^ including LV dilation and wall thinning, and eventual heart failure in many patients.^6^

MMP inhibition by systematic administration of pharmacological MMP inhibitors (MMPi) has shown promise in attenuating LV dilatation and reducing infarct size in MI and heart failure models.^7−9^ However, these drugs face challenges upon clinical translation. First, many of these molecules have poor water solubility and short half-lives (24–48 h post-administration). Therefore, they are unable to achieve effective inhibitory concentrations in the infarcted heart.^9,10^ Second, their repetitive dosing and nontargeted systemic delivery have led to off-target side effects in clinical trials, such as joint pain and stiffness associated with musculoskeletal syndrome.^11,12^ To address these problems, hydrogels have been used as controlled delivery platforms for MMPi biologics to improve targeting.^13,14^ The advantages of hydrogels include their potential for long-term retention in the infarct. However, the requirement of injection directly into the injured myocardium prevents their use during the acute phase of MI, given the risk of cardiac rupture and arrthymias.^15,16^ Nanoparticle (NP)-based therapeutics are an alternative delivery platform that can be administrated via minimally invasive intravenous (IV) injection and extravasate into the infarct through the highly permeable vasculature present acutely post-MI.^17,18^ Active targeting can also be achieved through NP surface functionalization,^19^ such as incorporating targeting moieties for upregulated angiotensin-1 receptor,^20,21^ P-selectin,^22^ and various mitochondrial targets.^23,24^ Despite this, NPs still face the risk of fast clearance (within hours to days post-injection) due to leakage from the infarct and opsonization, wherein biomolecules occlude the surface of particles and subsequently inhibit interaction with tissue-selective receptors.^25^ In light of this, a drug delivery platform that can be administered minimally invasively and can then selectively accumulate in the infarct for long-term retention is highly desirable.

Previously, our group developed MMP-responsive NPs that successfully targeted and were retained in the infarcted heart for up to 28 days following systematic administration.^26^ These materials were made from peptide-polymer amphiphiles (PPAs) where an inert hydrophobic block was followed by a hydrophilic block of MMP-2/MMP-9-cleavable peptides. Following IV injection, these NPs exited the leaky vasculature and were physically trapped in the infarcted region of the heart due to MMP-induced peptide cleavage, which altered the hydrophilic-to-hydrophobic ratio, and triggered subsequent material aggregation. This MMP-directed active assembly prevented the material from leaking out into the bloodstream, enabling long-term retention.^27,28^ In this work, we aimed to evaluate the ability of this platform to act as a targeted drug delivery system by conjugating a small-molecule MMP inhibitor (MMPi) drug, PD166793, to the polymer backbone for enhanced delivery and therapeutic retention in the infarct.^8^ PD166793 has been shown to significantly reduce MMP activity and LV dilation while preserving systolic function in a porcine MI model,^29^ but the requirement of repeated oral dosing to achieve a therapeutic outcome in combination with its poor water solubility has prevented its progression in clinical trials.^8,30^ We hypothesized that packaging this drug into enzyme-responsive NPs would allow for targeted delivery to the acute MI. The drug would then be shielded in the core of the NPs before exposure and release following MMP-induced peptide cleavage leading to aggregation in the infarct. As a first step toward the development of this therapeutic, in this study, we set out to determine whether PD166793-conjugated MMP-responsive micellar NPs would undergo MMP-mediated aggregation and targeting *in vivo* as well as to evaluate initial cytocompatibility and bioactivity.

## 2 Experimental Section

### 2.1 Materials and Animals

Organic solvents, including dimethyl formamide (DMF) and diethyl ether, were purchased from Fisher Scientific and used without purification. Chemical reagents were acquired from commercial vendors, including Sigma-Aldrich, Thermo Fisher Scientific, and Cambridge Isotope Laboratories, Inc., and used as received. The third-generation Grubbs catalyst (IMesH_2_)(C_5_H_5_N)_2_(Cl)_2_Ru = CHPh (G3),^31^*N*-hydroxysuccinimide-functionalized norbornene (NorNHS),^26^ and rhodamine-labeled terminating agent (Rho-TA)^31^ were prepared as previously described. Flash column chromatography was performed using silica gel 60 (40–63 μm, 230–400 mesh, 60 Å) purchased from Fisher Scientific. Analytical thin-layer chromatography (TLC) was carried out on silica gel 60G F254 glass plates purchased from EMD Millipore and visualized by observation of fluorescence under ultraviolet light and staining with KMnO_4_ as a developing agent. Dulbecco’s phosphate-buffered saline (without Ca^2+^, Mg^2+^) was purchased from Corning. Esterase from porcine liver was acquired from Sigma-Aldrich in the form of a lyophilized powder (≥15 U/mg solid). Thermolysin was acquired from Promega, as a lyophilized powder. Transmission electron microscopy (TEM) was performed on 400-mesh carbon grids purchased from Ted Pella, Inc. Murine L929s were obtained from Millipore. The alamarBlue reagent was ordered from Thermo and the MMP activity assay kit was purchased from Amplite. Female Sprague–Dawley rats were purchased from Envigo. Antibodies were acquired from Dako (anti-α-SMA), Thermo Fisher (Alexa Fluor-647), and Vector Laboratories (isolectin). Immunofluorescently stained slides were imaged using an upright Zeiss fluorescent microscope for 10–20× images and a Zeiss LSM confocal microscope for 63x magnification images. H&E stained slides were imaged on an Aperio ScanScope CS2 brightfield slide scanner.

### 2.2 Synthesis of Small-Molecule MMP Inhibitor PD166793 (MMPi)

MMPi was prepared following the modified procedures reported by O’Brien et al.^32^

#### 2.2.1 4′-Bromobiphenyl-4-sulfonic Acid

To a stirred solution of 4-bromobiphenyl (9 g, 38.6 mmol) in chloroform (80 mL), chlorosulfonic acid (5.4 g, 46.33 mmol) was added dropwise at room temperature (r.t). During the addition, precipitation was observed. The reaction mixture was stirred at room temperature for 5 h. Afterward, the white precipitate was collected by filtration, washed with cold chloroform, and oven-dried at 40 °C to a constant weight to give the final product (10.47 g, 87%). ^1^H NMR (500 MHz, Deuterium Oxide) δ 7.84–7.76 (m, 2H), 7.61–7.54 (m, 2H), 7.51–7.45 (m, 2H), 7.40–7.33 (m, 2H). ^13^C NMR (126 MHz, Deuterium Oxide) δ 142.20, 141.42, 138.04, 131.84, 128.67, 127.07, 126.00, 121.89.

#### 2.2.2 4′-Bromobiphenyl-4-sulfonyl Chloride

To a suspension of 4′-bromobiphenyl-4-sulfonic acid (5.94 g, 17.5 mmol) in dry DCM (300 mL), oxalyl chloride (11.12 g, 87.6 mmol) and a catalytic amount of dimethyl formamide (DMF, 64 mg, 0.88 mmol) were added. The reaction was heated to reflux and stirred overnight. The resulting solution was concentrated to yield a yellow solid, which was dissolved with EtOAc and washed with water. The organic layer was dried with brine and concentrated in vacuo to yield the product (5.2 g, 91%) as a yellow solid. ^1^H NMR (500 MHz, chloroform-*d*) δ 8.13–8.08 (m, 2H), 7.80–7.75 (m, 2H), 7.68–7.61 (m, 2H), 7.52–7.45 (m, 2H). ^13^C NMR (126 MHz, chloroform-*d*) δ 147.05, 143.19, 137.42, 132.46, 129.00, 128.02, 127.72, 123.82.

#### 2.2.3 PD166793 (MMPi)

To a mixture of l-valine, *t*-butyl ester hydrochloride (0.63 g, 3 mmol), and sulfonyl chloride (1.0 g, 3 mmol) in 1:1 THF/H_2_O (10 mL), triethylamine (NEt_3_, 0.61 g, 6 mmol) was added dropwise. The reaction was stirred at room temperature before diluting with EtOAc and washing with 1 M HCl. The organic layer was dried over MgSO_4_ and concentrated in vacuo. The crude was triturated with hexane and filtered to collect the white solid as the *tert*-butyl-protected PD166793 (1.2 g, 90%). ^1^H NMR (500 MHz, chloroform-*d*) δ 7.92–7.87 (m, 2H), 7.67–7.62 (m, 2H), 7.62–7.57 (m, 2H), 7.45–7.40 (m, 2H), 5.13 (d, *J* = 9.9 Hz, 1H), 3.66 (dd, *J* = 9.9, 4.5 Hz, 1H), 2.11–1.99 (m, 1H), 1.19 (s, 9H), 1.02 (d, *J* = 6.8 Hz, 3H), 0.86 (d, *J* = 6.9 Hz, 3H).

To a stirred solution of anisole (0.3 g) in trifluoroacetic acid (TFA, 5.6 mL) was added *tert*-butyl-protected PD166793. The reaction was stirred for 4 h at r.t and poured over ice (15 mL). The resulting precipitate was collected by filtration and washed with cold water before dried via lyophilization. The crude was recrystallized via EtOAC/Hex to yield the pure PD166793 as a pure white solid. ^1^H NMR (500 MHz, chloroform-*d*) δ 7.91–7.84 (m, 2H), 7.68–7.63 (m, 2H), 7.63–7.59 (m, 2H), 7.49–7.43 (m, 2H), 5.11 (d, *J* = 9.9 Hz, 1H), 3.82 (dd, *J* = 9.9, 4.6 Hz, 1H), 2.09 (pd, *J* = 6.9, 4.6 Hz, 1H), 0.96 (d, *J* = 6.8 Hz, 3H), 0.85 (d, *J* = 6.8 Hz, 3H). ^13^C NMR (126 MHz, chloroform-*d*) δ 174.36, 144.56, 138.54, 138.04, 132.26, 128.90, 127.90, 127.44, 123.10, 60.51, 31.42, 19.06, 17.06. ESI-MS: calculated for C_17_H_18_BrNO_4_S [M + H]^+^ 412.01 and 414.01; found [M + H]^+^ 411.9 and 413.9.

### 2.3 Synthesis of MMPi-Functionalized Norbornene Monomer (NorMMPi)

#### 2.3.1 NorHex-Valine

To a stirred solution of Boc-Valine (1.5 g, 6.9 mmol) and NorHex (2.0 g, 7.6 mmol) in dry DCM (20 mL) in ice/water bath under N_2_, a solution of *N*,*N*′-dicyclohexylcarbodiimide (DCC, 1.57 g, 7.6 mmol) and 4-dimethylaminopyridine (DMAP, 169 mg, 1.38 mmol) in 2 mL of DCM was added dropwise. The reaction was allowed to warm to room temperature and stirred for 24 h. After filtration to remove the insoluble urea, the organic layer was washed with 1.0 M HCl and brine and dried over MgSO_4_. The crude was concentrated in vacuo and purified via column chromatography (40% EtOAc in Hex) to yield a viscous liquid as the Boc-protected product (2.8 g, 88%). ^1^H NMR (400 MHz, chloroform-*d*) δ 6.22 (t, *J* = 1.9 Hz, 2H), 4.96 (d, *J* = 9.1 Hz, 1H), 4.13 (dd, *J* = 9.2, 4.7 Hz, 1H), 4.10–3.97 (m, 2H), 3.44–3.35 (m, 2H), 3.21 (p, *J* = 1.7 Hz, 2H), 2.61 (d, *J* = 1.3 Hz, 2H), 2.05 (h, *J* = 6.7 Hz, 1H), 1.63–1.42 (m, 5H), 1.41 (s, 9H), 1.34–1.20 (m, 4H), 1.18–1.11 (m, 1H), 0.89 (d, *J* = 6.9 Hz, 3H), 0.82 (d, *J* = 6.9 Hz, 3H).

To 8.5 mL of DCM were added NorHex-Boc-Valine (2.8 g, 6 mmol) and TFA (2.8 mL). The reaction mixture was stirred overnight before concentrating in vacuo. The residue was diluted with DCM, washed with NaHCO_3_, water, and brine, and dried over MgSO_4_ to yield the product (2.0 g, 92%). ^1^H NMR (400 MHz, chloroform-*d*) δ 6.30 (t, *J* = 1.9 Hz, 2H), 4.11 (td, *J* = 6.6, 2.7 Hz, 2H), 3.53–3.40 (m, 2H), 3.35–3.21 (m, 3H), 2.69 (d, *J* = 1.4 Hz, 2H), 2.03 (heptd, *J* = 6.9, 4.9 Hz, 1H), 1.70–1.47 (m, 8H), 1.45–1.28 (m, 4H), 1.23 (dt, *J* = 9.8, 1.6 Hz, 1H), 0.99 (d, *J* = 6.8 Hz, 3H), 0.91 (d, *J* = 6.9 Hz, 3H).

#### 2.3.2 NorMMPi

To a mixture of NorHex-Valine **5** (1.2 g, 3.3 mmol) and 4′-bromobiphenyl-4-sulfonyl chloride (1.09 g, 3.3 mmol) in THF (11 mL), triethylamine (NEt_3_, 0.67 g, 6.6 mmol) was added dropwise. An immediate formation of a white precipitate was observed. The reaction was stirred at room temperature for 5 h before the precipitate was filtered. The filtrate was diluted with DCM and washed with 1.0 M HCl. The organic layer was dried over MgSO_4_ and concentrated in vacuo to afford a white solid as the PD166793-conjugated norbornene NorMMPi (0.9 g, 64%). ^1^H NMR (500 MHz, chloroform-*d*) δ 7.91–7.86 (m, 2H), 7.68–7.64 (m, 2H), 7.63–7.58 (m, 2H), 7.49–7.43 (m, 2H), 6.28 (t, *J* = 1.9 Hz, 2H), 5.26 (d, *J* = 10.0 Hz, 1H), 3.85–3.71 (m, 3H), 3.41–3.35 (m, 2H), 3.27 (t, *J* = 1.9 Hz, 2H), 2.67 (d, *J* = 1.3 Hz, 2H), 2.05 (pd, *J* = 6.8, 5.0 Hz, 1H), 1.53–1.34 (m, 5H), 1.19 (dd, *J* = 7.2, 3.3 Hz, 5H), 0.98 (d, *J* = 6.8 Hz, 3H), 0.88 (d, *J* = 6.8 Hz, 3H). ^13^C NMR (126 MHz, chloroform-*d*) δ 178.11, 178.09, 171.30, 144.30, 138.74, 138.03, 137.83, 132.28, 128.80, 128.00, 127.30, 123.06, 65.39, 61.15, 47.82, 45.18, 42.73, 38.32, 31.70, 28.13, 27.46, 26.32, 25.19, 19.03, 17.41.

### 2.4 Synthesis of MMP-2/MMP-9-Cleavable Peptide

Peptides with the amino acid sequence, GPLGLAGGWGERDGS, were synthesized on rink amide 4-methyl benzylhydrylamine (MBHA) resin via Fmoc-based solid-phase peptide synthesis similarly as described by Nguyen et al.^26^ The underlined amino acids represent an MMP-2 and -9 recognition sequence. The resin was allowed to swell in DMF for 2 h. Fmoc deprotection was performed by agitating resin in 20% 4-methylpiperidine in DMF for 5 draining and repeating this procedure for another 15 min. Amino acid couplings were carried out for 45 min per amino acid using *N*,*N*,*N*′,*N*′-tetramethyl-O-(1H-benzotriazol-1-yl)uronium hexafluorophosphate (HBTU) and N,N-diisopropylethylamine (DIPEA) (resin/amino acid/HBTU/DIPEA 1:3:3:6). Final peptides were cleaved from resin by treatment with trifluoracetic acid (TFA), triisopropyl silane (TIPS), dithiothreitol (DTT), and water (TFA/TIPS/DTT/H_2_O 88% v/v:2% v/v:5% w/v: 5% w/w) for 2 h. Peptides were then precipitated in cold diethyl ether and centrifuged at 10,000 rpm for 10 min. This procedure was repeated twice. The precipitated peptide was dried in vacuo to give the crude product. The purity of the crude was examined via reversed-phase high-pressure liquid chromatography with a UV detector (HPLC-UV). Buffer A (0.1% TFA in H_2_O) and Buffer B (0.1% TFA in ACN) were used as the mobile phase. The HPLC run was performed using a 15–40% B gradient in 30 min. The peptide signal eluted at 14 min when detected at 214 nm. The same gradient was adopted for peptide purification using preparative HPLC. The purified peptide was dried via lyophilization, giving a white solid. ESI-MS: calculated for C_61_H_94_N_20_O_20_ [M + H]^+^ 1427.7; found [M + H]^+^ 1427.7 and [M + 2H]^2+^ 714.4. The responsive l-MMP peptide, nonresponsive d-MMP peptide, and the cleaved sequence LAGGWGERDGS were all synthesized and purified by this method.

### 2.5 Representative Procedures for Peptide-Polymer Amphiphile (PPA) Synthesis

Peptide-polymer amphiphiles (PPAs) were prepared via graft-to ring-opening metathesis polymerization (ROMP) as shown in Figure 2.

#### 2.5.1 Synthesis of Block Copolymer via ROMP

DMF was freeze–pump–thawed over three cycles. All of the monomers and the modified third-generation Grubbs catalyst **G3**, (IMesH_2_)(C_5_H_5_N)_2_(Cl)_2_Ru = CHPh, were weighed separately in vials charged with stirring bars. The monomers and DMF were then loaded into a glovebox under N_2_. To a stirring solution of **G3** (3.28 mg, 1.0 equiv) in DMF, a mixture of phenyl norbornene (NorPh, 14.82 mg, 13 equiv) and NorMMPi (20.72 mg, 7 equiv) in DMF was added under N_2_ to yield the random block copolymer NorPh_13_-*co*-NorMMPi_7_. After 45 min, an aliquot (∼10 μL) was removed for SEC-MALS analysis to confirm complete monomer consumption (Table S1). The chain extension was achieved by the addition of N-hydroxysuccinimide-functionalized norbornene (NorNHS, 5.3 mg, 5 equiv). After 1 h, the rhodamine-labeled terminating agent (Rho-TA, 10 mg, 2 equiv)^31^ in DMF was added to quench the catalyst. After 30 min of stirring, the reaction was moved out of the glovebox. The polymers were precipitated in cold diethyl ether and collected by centrifugation at 10,000 rpm for 10 min. The same procedures were repeated three times. The final polymers were dried in vacuo, and the molecular weights were analyzed by SEC-MALS (Table S1).

#### 2.5.2 Peptide Conjugation via Post-Polymerization Modification

To polymer (46 mg, 1 equiv) dissolved in DMF, MMP peptide substrate (67.7 mg, 10 equiv regarding polymer, 2.0 equiv regarding NHS) was added, giving a concentration of 50 mg/mL peptide. 5 μL of the solution mixture was removed and dissolved in 245 μL of 15% Buffer B in A for HPLC-UV analysis as the *t* = 0 reference (detection at 214 nm). *N*,*N*-Diisopropylethylamine (DIPEA, 30.7 mg, 50 equiv) was then added to the reaction mixture and stirred at room temperature overnight. After confirming peptide consumption by HPLC-UV (sample prepared similarly as *t* = 0), the peptide-polymer amphiphiles (PPAs) were precipitated in cold diethyl ether and collected via centrifugation. The resulting crude was dissolved in 1:1 DMSO: water and dialyzed against water in a SnakeSkin Dialysis Tubing (10K MWCO). Three water changes were performed in 48 h, and the resulting solution was lyophilized dry to give the pure PPAs. The molecular weight of the dried PPAs was analyzed by SEC-MALS (Table S1).

Similar procedures were used to prepare the nonfluorescent peptide-polymer amphiphiles with maximum MMPi loading **PPA**_**Max**_ and control **PPA**_**C**_ (Table S2). Instead of using Rho-TA, the reactions were terminated with ethyl vinyl ether (EVE).

### 2.6 Calculation of PPA Drug Dosage

Due to limited water solubility, PD166793 has never been delivered intravenously in an animal model. In a previous report, a plasma concentration of 116 ± 11 μmol/L was shown to attenuate LV dilation and dysfunction in a rat model of progressive heart failure following 4 months of 5 mg·kg^–1^·day^–1^ PO in chow.^33^ Drug dosage in PPAs and subsequently in the NPs is summarized in Table S3. The dosage calculation is based on the following assumptions and equations:

300 nmol polymer in 1 mL of DPBS for injection

250 g average rat body weight with 16 mL of total blood volume

17 mL total body fluid volume (16 mL blood + 1 mL NP solution)
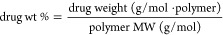

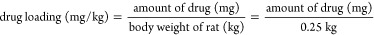

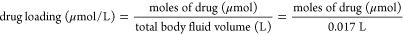


### 2.7 NP Formulation

PPAs were dissolved at 3 mg/mL in DMSO. 1× Dulbecco’s phosphate-buffered saline (DPBS, without Ca^2+^ and Mg^2+^) was added via a syringe pump at the speed of 100 μL/h until reaching 30% DPBS in DMSO (v/v). The solution was left stirring overnight and transferred into SnakeSkin Dialysis Tubing (10k MWCO) to dialyze against DPBS for 48 h with three buffer changes. The resulting solution was filtered through a 0.22 μm PES membrane to remove any large aggregates. The polymer concentration of the filtered solution was confirmed by UV absorbance from rhodamine. The NP solution was concentrated by spin centrifugation to give 300 μM regarding the polymer. Similar procedures were used to prepare the maximum drug-loaded NPs (**NP**_**Max**_) and control NPs (**NP**_**C**_), whereas the concentrations were determined by the initial mass-to-volume ratio.

### 2.8 *In Vitro* Enzyme-Induced Aggregation

NPs (100 μM, with respect to polymer) were treated with Zn-metalloproteinase thermolysin (1 μM), an MMP alternative with improved *in vitro* activity, or DPBS for 24 h at 37 °C in 1X DPBS. The resulting NP solutions were analyzed by dynamic light scattering (DLS) and transmission electron microscopy (TEM) to examine the change in morphology. For the TEM samples, 5 μL of sample was applied to a 400-mesh carbon grid (Ted Pella, Inc.) that had been glow-discharged for 15 s. 5 μL of 2 wt % uranyl acetate solution was then applied and wicked away after 30 s of staining.

### 2.9 *In Vitro* MMP Activity Assay

Due to the poor water solubility of PD166793, a stock solution of free drug and NorMMPi (12 mM) was prepared in DMSO. 12 mM Ethylenediaminetetraacetic acid (EDTA) in DMSO was also prepared as the negative control due to its strong MMP inhibiting effect by chelating to the Zn cofactor at the MMP active site.^34^ To 1 μL of MMP-9 stock solution (38.5 μM stock, 0.5 μM final), 1.5 μL of DMSO/NorMMPi/free MMPi/EDTA (12 mM stock, 235 μM final) was added and incubated at 37 °C for 5 min. 75 μL of MMP peptide (500 μM) in DPBS was then added and incubated at 37 °C for 24 h before HPLC-UV analysis. The instrument used 0.1% trifluoroacetic acid (TFA) in water as Buffer A and 0.1% TFA in acetonitrile as Buffer B. For each HPLC sample, 50 μL reaction crude was diluted with 150 μL of DPBS. 40 μL of the resulting solution was injected and ran over the gradient of 0–40% Buffer B in 30 min (Figure S1). The percent peptide cleavage was quantified and used as the criterion to evaluate MMP activity.

### 2.10 Esterase-Catalyzed PD166793 Release

A stock solution of MMPi and NorMMPi was prepared in DMSO at 13 mM. 5 μL of esterase (26.4 μM), 5 μL of NorMMPi, and 100 μL of DPBS were mixed in Eppendorf tubes, giving the final concentration of 1.2 μM esterase: 600 μM NorMMPi. The solution was incubated at either 25 or 37 °C for 24 h. MMPi and NorMMPi at 600 μM in DPBS were also prepared from the stock as controls. The samples were diluted to 220 μL for HPLC-UV analysis (Figure S2). Condition: 10 μL injection, 40–80% Buffer B over 30 min.

### 2.11 Surgical Procedures and IV Injection

All procedures in this study were approved by the Committee on Animal Research at the University of California, San Diego, and the Association for the Assessment and Accreditation of Laboratory Animal Care. Female Sprague–Dawley rats (225–250 g) underwent ischemia-reperfusion (IR) procedures via left thoracotomy and temporary occlusion of the left anterior descending artery for 35 min.^35^ One day post-MI, the animals were anesthetized using isoflurane and arbitrarily assigned to IV injection of either 1 mL of MMPi NPs (300 μM) or saline and harvested at 1 day (*n* = 2) or 6 days (*n* = 3) post-injection. The animals were euthanized via overdose of pentobarbital (200 mg/kg), and the heart, kidney, spleen, lungs, and liver were collected for histological analysis.

### 2.12 Histology and Immunohistochemistry

Following euthanasia, hearts were dissected and freshly frozen in OCT (optimal cutting temperature) compound for cryosectioning. Hearts were stained with hematoxylin and eosin to visualize the infarcted region of the heart. Slides were scanned on an Aperio ScanScope CS2 brightfield slide scanner. Additional heart sections were stained with anti-α-SMA (1:75 dilution, Dako), Alexa Fluor-647 (1:500 dilution, Thermo Fisher), and isolectin (1:75 dilution, Vector Laboratories) to visualize large arterioles and capillaries.

### 2.13 Cytocompatibility of MMPi NPs and Free MMPi

Murine fibroblast cells (L929) were seeded into a 96-well plate and left to adhere overnight. Following cell adhesion, MMPi **NP**_**Max**_ were diluted with sterile PBS to generate concentrations ranging from 41 to 9 μM and were added to the media with PBS and zinc diethyldithiocarbamate (ZDEC) at a concentration of 47 mM serving as positive and negative controls, respectively. Treated cells were then incubated for 24 h at 37 °C before performing an alamarBlue assay to evaluate their metabolic activity.

To compare the cytocompatibility of MMPi NPs and free MMPi, L929s were again plated and allowed to adhere overnight. The cells were then treated with MMPi **NP**_**Max**_ or an equivalent concentration of free MMPi ranging from 1200 to 9.375 μM. PBS was used as a positive control, and DMSO was used to control the solvent used to dissolve the free drug. After 24 h of treatment incubation at 37 °C, an alamarBlue assay was run on treated cells to evaluate their metabolic activity.

### 2.14 Assessment of MMP Activity *In Vitro*

Adherent murine fibroblasts (L929s) were plated and allowed to adhere overnight in a 96-well plate at a seeding density of 16,000 cells/well. Media from the plated cells was collected and treated with either MMPi **NP**_**Max**_ (14.28 μM with respect to polymer, 285 μM with respect to drug), free MMPi (285 μM), empty NPs (**NP**_**C**_), PBS, or DMSO. The free drug concentration was calculated to match that loaded in the **NP**_**Max**_. Following 20 min of treatment, an MMP cleavage fluorescent FRET peptide (Amplite) was added to track MMP activity over time following the manufacturer’s instructions. The plate was then incubated at 37 °C and data points were taken every 20 min for 3 h.

### 2.15 Macrophage Recruitment Following MMPi NPs Administration

In animals harvested at 7 days post-MI, heart sections were stained and quantified for general macrophage presence. To identify macrophages, sections were stained with anti-CD68 (1:100 dilution, BioRad) and goat anti-mouse IgG (H+L) conjugate (1:500 dilution, BioRad) and developed using a DAB solution kit (Thermo Fisher). Images were taken using a scanning light microscope (Aperio ScanScope CS2).

## 3 Results and Discussion

### 3.1 NorMMPi Serves as a Prodrug and Bioactive PD166793 Can Be Released via Proteolysis

**Figure 1:**
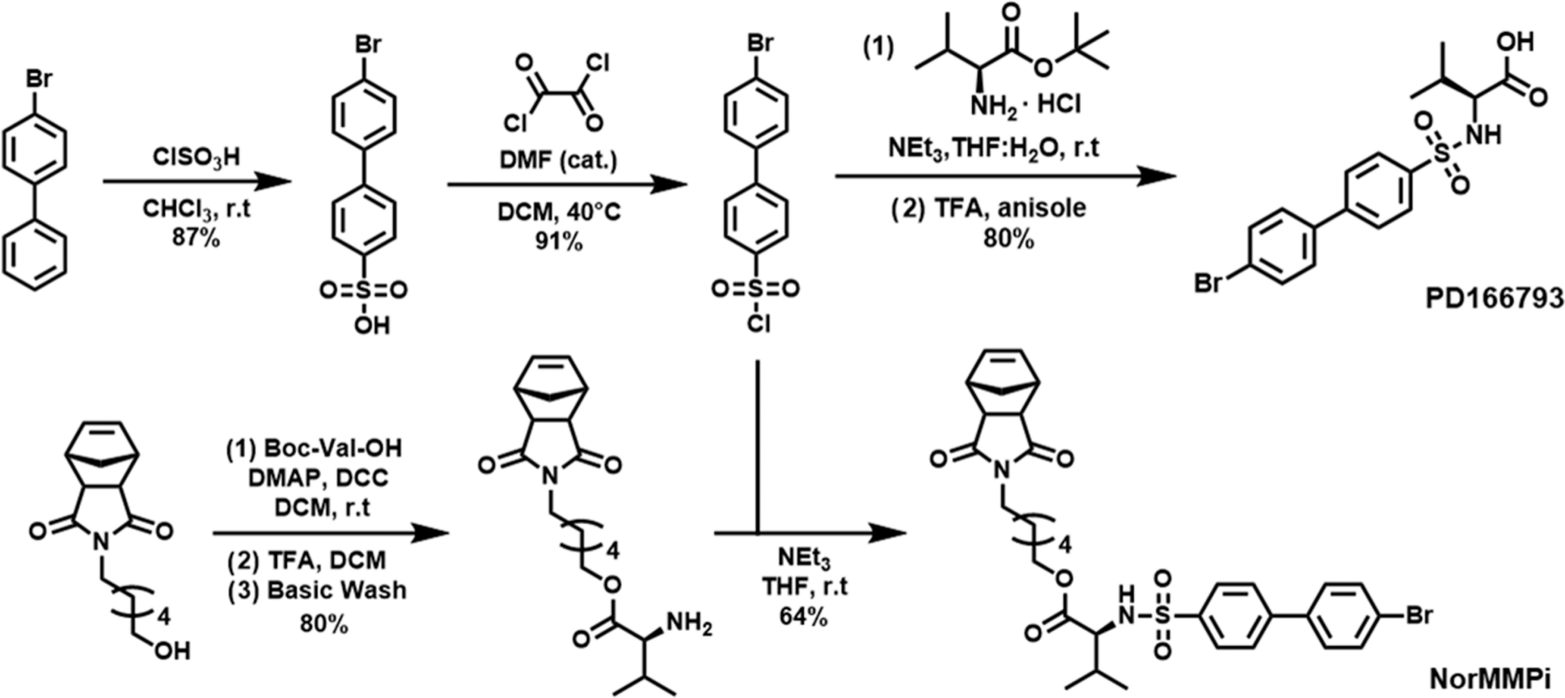
Synthesis of MMP inhibitor PD166793 and its norbornene functionalized monomer NorMMPi.

To start the investigation, the MMP inhibitor (MMPi) PD166793 and its functionalized norbornene monomer (NorMMPi) were synthesized as shown in Figure 1. Their inhibitory effects against MMP were then examined. Briefly, MMPs were incubated with either PD166793 or NorMMPi before the addition of a peptide substrate GPLGLAGGWGERDGS (MMP cleavage site underlined).^26^ The cleavage of peptide was monitored via high-performance liquid chromatography coupled with a UV detector (HPLC-UV) and used as the criteria to evaluate MMP activity. Two control groups, including DMSO (i.e., no MMP inhibition) and ethylenediaminetetraacetic acid (EDTA), a strong metal chelator (i.e., full MMP inhibition) were used. As compared to the results from control groups, free PD166793 blocked 70% of MMP activity, while no MMP inhibition was observed upon the NorMMPi treatment (Figure S1). This result indicates that NorMMPi shields the carboxylic acid from interaction with MMP catalytic center, which agrees well with the previously reported drug mechanism of action.^8^ To confirm the bioactive PD166793 can be released from NorMMPi via ester bond cleavage, an esterase-mediated proteolysis assay was set up (Figure S2).^36^ PD166793 has a strong UV absorbance at 270 nm, which enabled us to monitor its release via HPLC-UV (Figure S2A,B). While NorMMPi showed good stability in buffer and did not display any HPLC signal under the running condition, we observed more than 90% PD166793 release after 24 h of incubation with esterase at 37 °C (Figure S2C). This result suggests that the drug in the NP core should be amenable for release upon exposure to the enzyme-rich infarct environment after MMP-induced aggregation.

### 3.2 PD166793-Loaded NPs (MMPi NPs) Maintained Enzyme Responsiveness

**Figure 2:**
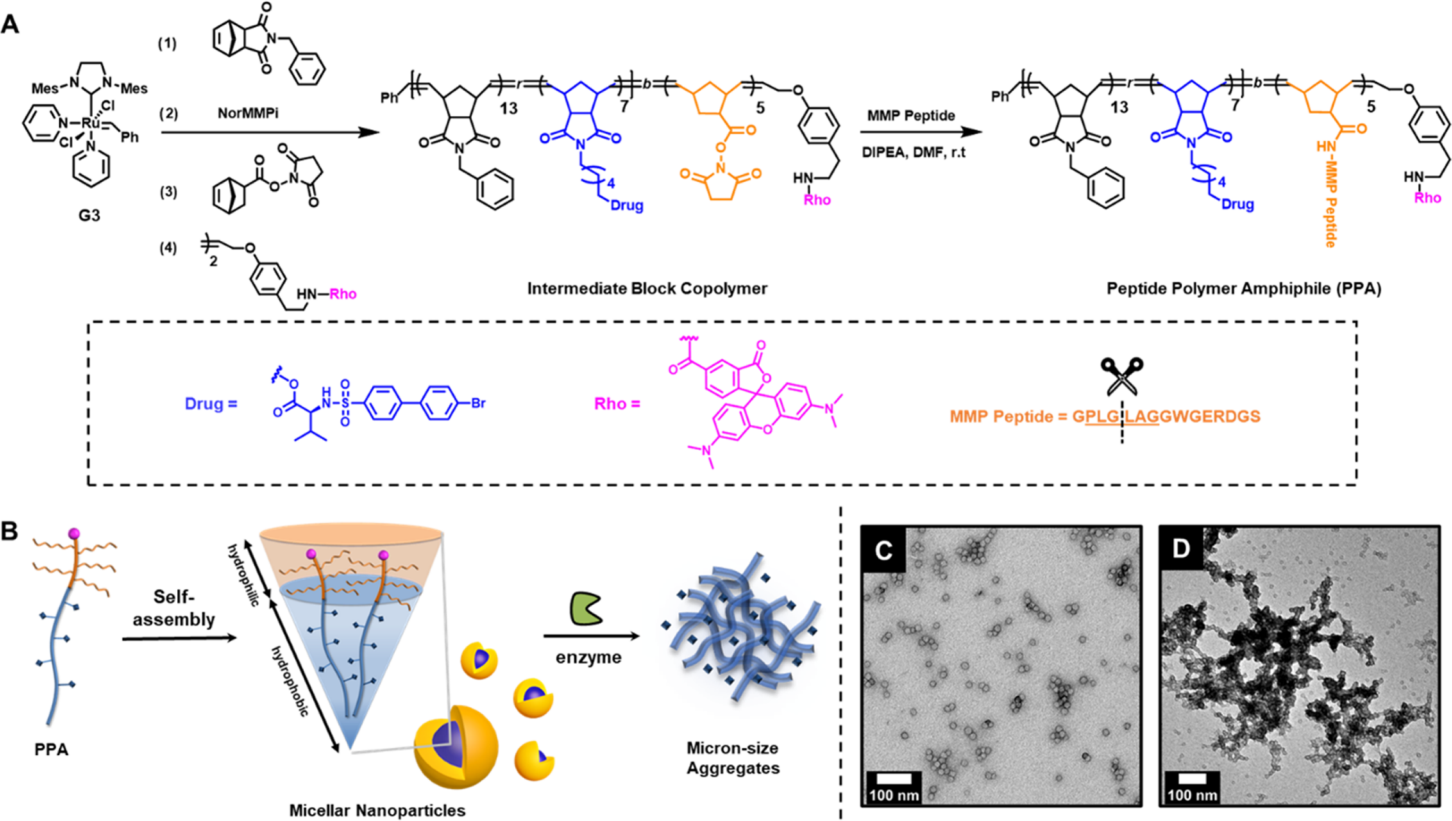
Synthesis of PD166793-loaded NPs and enzyme-induced morphology switch. (A) Rhodamine-labeled, PD166793-loaded peptide-polymer amphiphiles (PPAs) were prepared via a two-step procedure, including ring-opening metathesis polymerization (ROMP) to establish the polymer backbone and post-polymerization modification to conjugate the MMP peptide substrate. (B) Schematic representation of PPA assembly to form NPs followed by enzyme-induced microscale aggregate formation. Transmission electron microscopy images of (C) NPs and (D) aggregates formation post-enzyme treatment.

Rhodamine-labeled, PD166793-loaded peptide-polymer amphiphiles (PPAs) (20% wt % drug) were prepared via graft-to ring-opening metathesis polymerization (ROMP) as shown in Figure 2A. The consumption of peptide was confirmed via HPLC-UV, and the growth of polymer molecular weight was confirmed by size-exclusion chromatography coupled with multi-angle light scattering (SEC-MALS) (Figure S3, Table S1). The degree of polymerization (DP) of each block was optimized, targeting a ratio of 13:7 phenyl norbornene (NorPh) to NorMMPi to provide a PD166793 plasma level (assuming 100% drug release after injection of 300 nmol PPA in the rat model) similar to the effective concentration of 100 μmol/L.^26,33^ Calculations can be found in the Supporting Information (Table S3).

By solvent switch from DMSO to Dulbecco’s phosphate-buffered saline (DPBS), PPAs assembled into spherical micelles with a diameter of 15 nm as visualized by TEM (Figure 2B,C). To examine the enzyme responsiveness, the MMPi NPs were incubated with Zn-metalloproteinase thermolysin, which serves as a robust alternative to MMP but with higher *in vitro* activity, at 37 °C in DPBS (1 μM thermolysin: 100 μM polymer). The formation of micron-size assemblies was then detected by both TEM (Figure 2D) and dynamic light scattering (DLS) measurements (Figure S4). Therefore, this *in vitro* analysis allowed us to conclude that these PD166793-loaded NPs maintained good enzyme responsiveness.

### 3.3 MMPi NPs Localized to the Infarct in a Rat Acute MI Model

**Figure 3:**
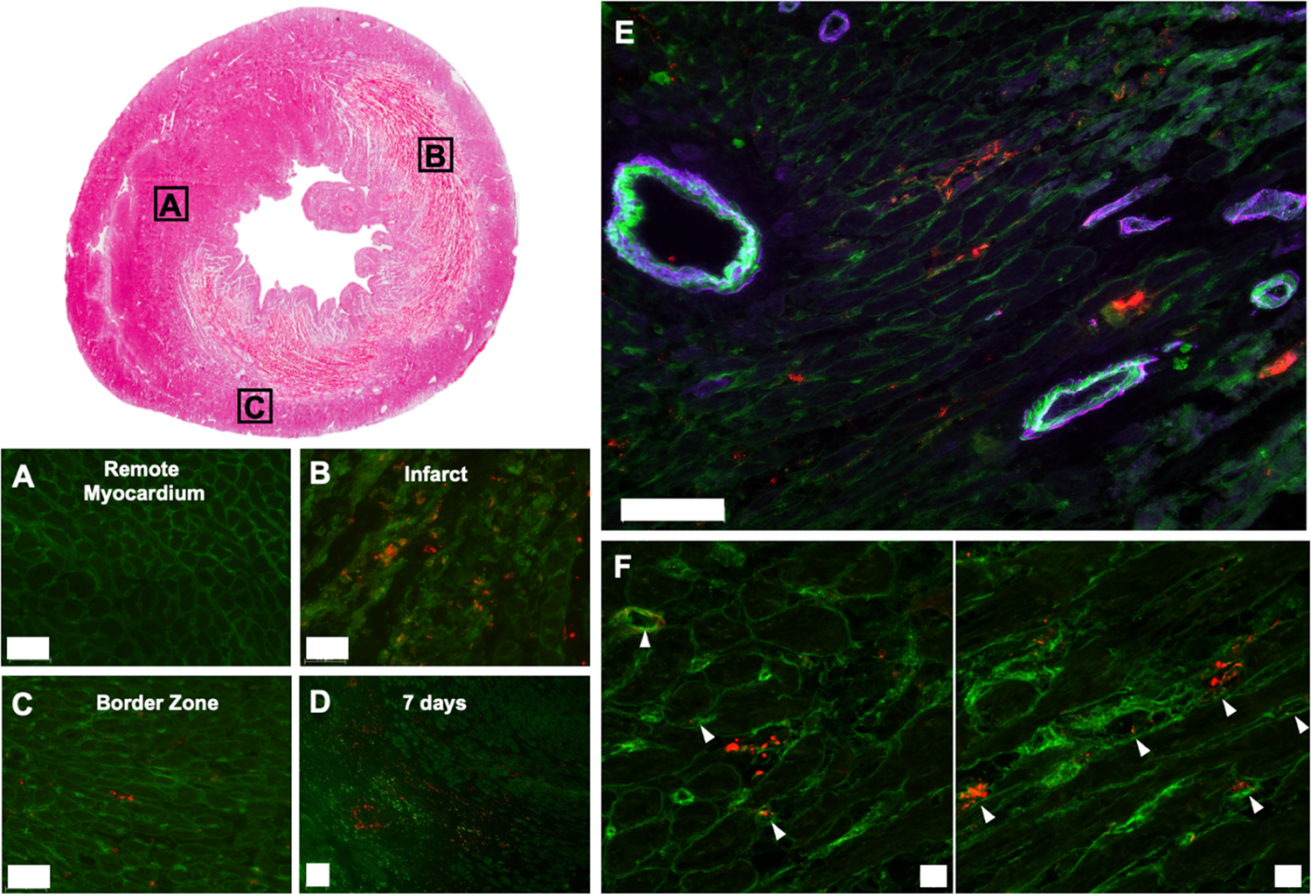
MMPi NPs extravasate from leaky vasculature, localize, and aggregate in acute MI. Following IV injection, rhodamine-labeled MMPi NP aggregates localized to the infarct (B) with some accumulation in the border zone (C) and no accumulation in the remote myocardium (A) (scale bars: 50 μm). (D) At 7 days post-injection, MMPi NP aggregates were still present in the infarct (scale bar: 100 μm). (E) MMPi NP aggregates did not obstruct the microvasculature (scale bar: 100 μm). (F) Confocal imaging showed a combination of extracellular deposition of MMPi NP aggregates as well as some aggregate formation at the endothelium in capillaries (identified by white arrows, scale bar: 10 μm). Red = NP aggregates, purple = α-SMA, green = isolectin.

MMPi NPs (300 μM regarding polymer) were administered via IV injection in a rat model one day post-MI (Figure 3). MMPi NP aggregates, as visualized by fluorescent microscopy, were found to accumulate in the infarct with limited retention in the border zone. No MMPi NP aggregates were observed in the remote myocardium (septum) or right ventricle (Figure 3A–C). This result demonstrates regioselective accumulation and retention of MMPi NP aggregates in the heart, which is consistent with their previously reported non-drug-loaded analogues.^26^ In addition, these MMPi NP aggregates remained visible in the infarct at 7 days post-injection (Figure 3D), suggesting long-term retention.

### 3.4 MMPi NPs Accumulated in the Infarcted Region

The proposed mechanism of aggregation and accumulation in acute MI involves extravasation of NPs from the leaky vessels to the heart where they will encounter upregulated extracellular MMPs and undergo peptide cleavage, aggregation, and accumulation. To confirm localization, smooth muscle cells and endothelial cells were stained with anti-α-smooth muscle actin (α-SMA) and isolectin for visualization, respectively. MMPi NPs were mostly found outside of the vasculature, and fluorescence microscopy images revealed that MMPi NPs did not block arterioles (Figure 3E). Through confocal microscopy, we observed MMPi NPs aggregation in the extracellular space as well as at the endothelium of some capillaries, which could be attributed to the premature aggregation caused by the MMP released from the endothelial cells^37^ (Figure 3F). Importantly, we observed that the vessel lumens remained open and unobstructed by the rhodamine-labeled NPs. We note that delivery of an MMPi not only to the extracellular matrix of the infarcted myocardium but also endothelial cells could increase the therapeutic effect as endothelial cells also produce MMPs during the acute phase of MI.^37^

### 3.5 Macrophage Density Was Not Impacted by MMPi NPs Administration

**Figure 4:**
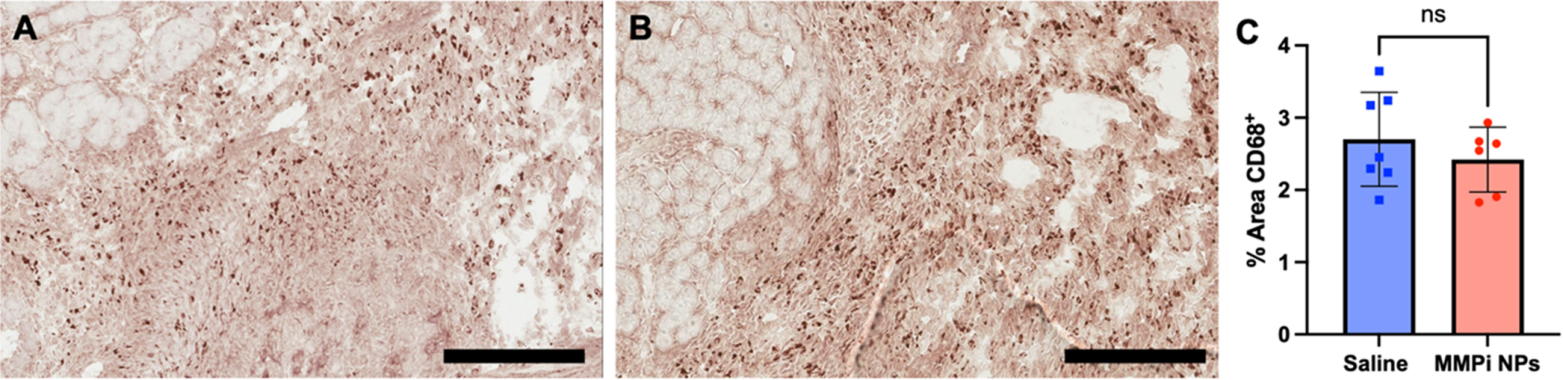
CD68 staining in the infarct at 7 days post-injection shows no sign of increased macrophage presence when comparing saline-injected (A) and NP-injected (B) hearts (scale bar: 200 μm). (C) Quantification of macrophage density.

In animals harvested at 7 days post-MI, heart sections were stained for macrophages (Figure 4). We observed no significant differences in macrophage density between saline and MMPi NPs-treated animals (Figure 4A–C). This allows us to conclude that our MMPi NPs did not increase the recruitment of macrophages into the infarcted region, which is a preliminary indicator of *in vivo* biocompatibility.

### 3.6 Maximally Loaded MMPi NPs Are Enzyme-Responsive, Cytocompatible, and Bioactive

**Figure 5:**
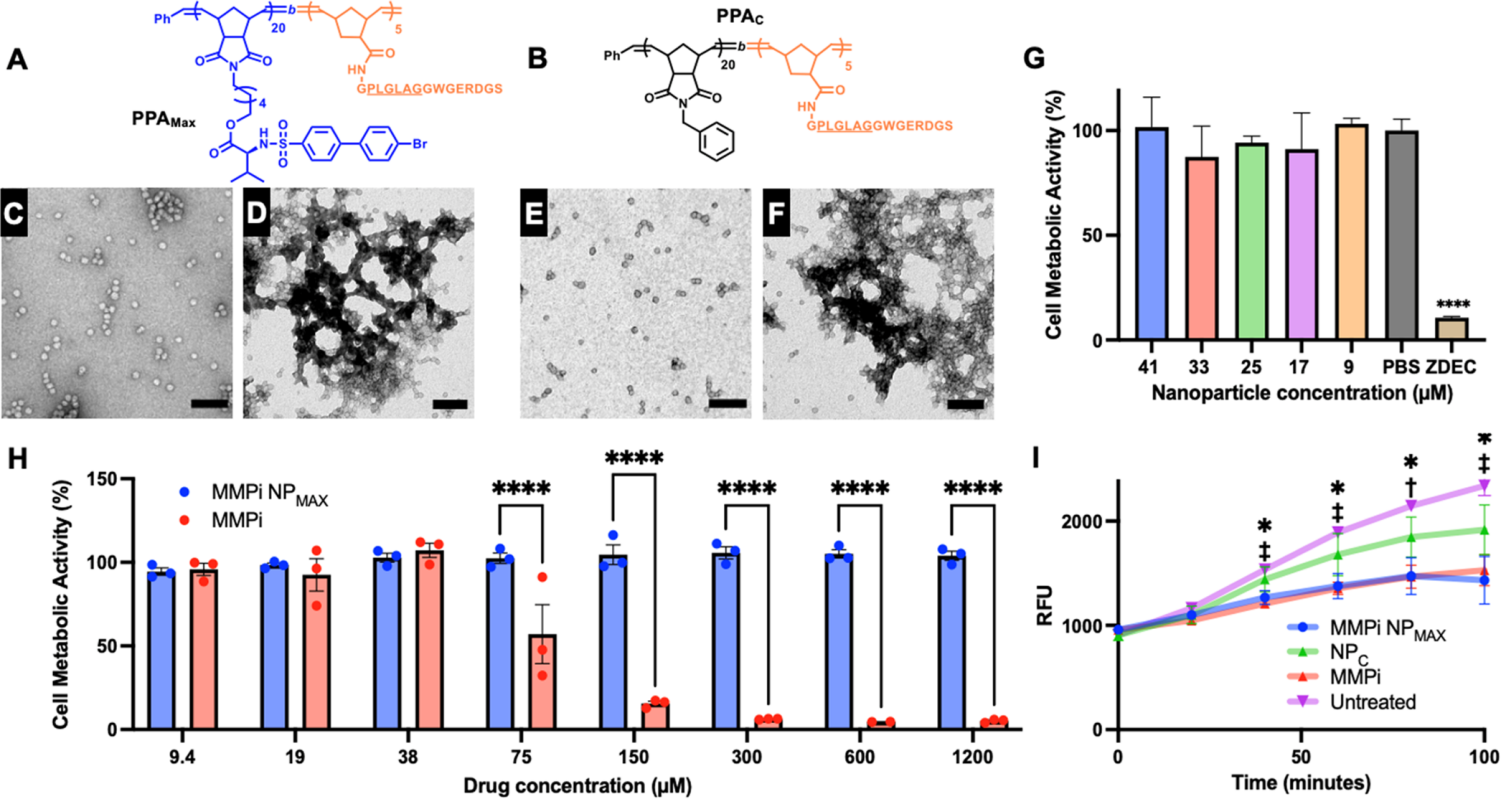
Enzyme responsiveness, cytocompatibility, and bioactivity analysis of maximally loaded MMPi NP_Max_. Max drug-loaded **PPA**_**Max**_ (A) and control **PPA**_**C**_ (B) still formed micellar NPs (C) and (E) of the same size as the original MMPi NPs (20 wt % drug). Additionally, **NP**_**Max**_ underwent a morphological switch into micron-scale assemblies after incubation with thermolysin (D), similar to their **NP**_**C**_ counterparts (F). (G) **NP**_**Max**_ does not significantly impact metabolic activity via alamarBlue assay compared to PBS. The metabolic activity of all of the treatment groups was normalized to the PBS control. Conversely, the negative zinc diethyldithiocarbamate (ZDEC) control, the international standard reference material for evaluating the *in vitro* cytotoxicity of polymer materials,^39^ shows a significant reduction in metabolic activity. (H) Treatment of L929s with free drug (MMPi) results in reduced metabolic activity at high concentrations, whereas **NP**_**Max**_ offers a protective effect by shielding the drug in the particle pore. (I) MMP activity is significantly decreased when treated with **NP**_**Max**_ compared to an untreated control (****P < 0.001 via 1-way ANOVA with Tukey’s post hoc test, *P < 0.05 **NP**_**Max**_ compared to untreated, ^†^*P* < 0.05 MMPi compared to untreated, ^‡^*P* < 0.001 MMPi compared to untreated via 2-way ANOVA with Tukey’s post hoc test).

In the interest of delivering the maximal amount of drug, we decided to evaluate the fully drug-conjugated polymer backbone for enzyme responsiveness, cytocompatibility, and bioactivity *in vitro* (Figure 5). To achieve this, **PPA**_**Max**_ with a DP of 20:5 (NorMMPi:peptide) was synthesized as described above (Figure S5A, Table S2) and formulated into **NP**_**Max**_ (Figure 5A,C). As compared to the previously discussed MMPi NPs (20% wt % drug), the drug loading was increased to 40% wt % for **NP**_**Max**_ (Table S3). **PPA**_**C**_ without drug incorporation (NorPh: peptide = 20: 5) was also prepared as a control (Figure S5B) and assembled into spherical micelles **NP**_**C**_ (Figure 5B,E). TEM analysis revealed that both **NP**_**Max**_ and **NP**_**C**_ had similar sizes as the 20% wt % drug MMPi NPs (15–20 nm in diameter) and underwent thermolysin-induced aggregation to form micron-scale assemblies (Figure 5D,F), suggesting that high drug loading did not affect enzyme responsiveness.

Next, we proceeded to evaluate the cytocompatibility of **NP**_**Max**_ by incubating it with L929 fibroblasts. L929s were used here as they are a standard cell line for establishing cytocompatibility in accordance with the UNI ISO 10993/2009 for cytotoxicity assays. We employed various **NP**_**Max**_ concentrations between 9 and 41 μM to account for the difference in particle circulating concentration (∼18 μM once diluted in the total blood volume of the rat^38^) as well as higher concentrations to account for the time prior to uniform distribution in the blood and potential enhanced concentration once they localize to the tissue. Results from an alamarBlue assay showed that **NP**_**Max**_ did not significantly alter L929 fibroblast metabolic activity compared to a positive PBS control at concentrations up to 41 μM (Figure 5G). To further compare the impact of free and shielded drug on cell metabolic activity, L929s were treated with free MMPi and **NP**_**Max**_ in parallel. Although no significant difference was noticed at low drug concentrations, we observed a significant decrease in cell metabolic activity after the treatment with free drug at high drug concentrations (75–1200 μM) compared to **NP**_**Max**_ (Figure 5H). This drug concentration correlates to an NP concentration ranging from 3.75 to 60 μM, which aligns with the aforementioned physiologically relevant concentration of MMPi NPs in the body and infarcted region. This result demonstrates preliminary safety of our MMPi-loaded NPs, suggesting a higher drug dose could be safely administered when it is packaged into our NP platform compared to free drug delivery.

Finally, we sought to confirm the bioactivity of MMPi conjugated to the polymer backbone in the maximally loaded NPs **NP**_**Max**_. In a time-course experiment where fluorescent readings were performed every 20 min to detect signal from a fluorescent MMP cleavage FRET peptide, we observed that both **NP**_**Max**_ and free MMPi were capable of significantly inhibiting MMP activity within 40 min of incubation compared to a PBS control by 17.4 and 21.3%, respectively (Figure 5I). From this, we conclude that packaging the small-molecule drug does not negatively impact the bioactivity of PD166793. Given the fact that PD166793 only becomes bioactive post-ester bond cleavage, this result indicates that there is some rapid drug release from **NP**_**Max**_. This quick release could be beneficial for efficient therapeutic delivery during the acute phase of MI and may improve local cytocompatibility when compared to free drug administration at a comparable dose as evidenced by the *in vitro* data (Figure 5H,I). Future studies could also evaluate and compare the biocompatibility and biodistribution of this system to encapsulate the drug in the nanoparticle core. Knowing that the drug is still bioactive and that a higher dose may be tolerable when conjugated to the polymer backbone, it is possible that utilizing the presented NP platform could allow for safe delivery of high concentrations of PD166793, thus eliminating the need for excessive repeat doses, although further *in vivo* studies will be needed.

## 4 Conclusions

In summary, we have developed a novel enzyme-responsive NP therapeutic delivery system for acute MI. Using ROMP, different dosages of a small-molecule MMP inhibitor PD166793 can be loaded into peptide-polymer amphiphiles through a hydrolyzable ester linkage, and subsequently into the core of micellar NPs. The resulting NPs demonstrated regioselective accumulation and retention for up to 7 days in the infarcted heart after IV administration in a rat MI model. Further *in vitro* studies revealed that incorporation of this drug onto the polymer backbone and subsequently into the core of NPs mitigates its cytotoxicity while maintaining its bioactivity, allowing for toleration of higher dose. Future studies could investigate the drug release profile, biodistribution, and clearance of this nanoparticle platform *in vivo*. This study demonstrates initial proof of concept for the conjugation of an MMPi to MMP-responsive NPs. Since any therapeutic that is amenable to chemical conjugation can be potentially introduced in the form of a monomer,^40−43^ we envision this targeted NP platform to be a generalizable approach for drug delivery and the treatment of other inflammatory diseases. Future studies will continue to explore the efficacy of these polymeric nanomaterials as therapeutic carriers during the acute phase of myocardial infarction.

## Supporting Information Available

SEC-MALS characterization of original PPA and intermediates; SEC-MALS characterization of PPA_Max_, PPA_C_, and intermediates; summary of PPA drug dosage; HPLC-UV analysis of MMP-cleavable peptide post incubation with MMP-9 treated with DMSO, NorMMPi, free PD166793, or EDTA; esterase-catalyzed PD166793 release from NorMMPi; synthesis of PD166793-incorporated peptide-polymer amphiphile; DLS analysis of PD166793-loaded nanoparticles (NPs) before and after thermolysin treatment; synthesis of PPA_Max_ and PPA_C_; UV absorbance of Rho-TA and calibration curve at 548 nm; ^1^H and ^13^C NMR spectra of 4′-bromobiphenyl-4-sulfonic acid in D_2_O; ^1^H and ^13^C spectra of 4′-bromobiphenyl-4-sulfonyl chloride in CDCl_3_; ^1^H NMR spectrum of *t*-butyl-ester-protected PD166793 in CDCl_3_; ^1^H and ^13^C NMR spectra of PD166793 in CDCl_3_; HSQC spectra of PD166793 in CDCl_3_; ^1^H NMR spectra of NorHex-Boc-Valine and NorHex-Valine in CDCl_3_; ^1^H and ^13^C NMR spectra of NorMMPi in CDCl_3_; and HSQC spectra of PD166793 in CDCl_3_ (PDF)

## Abbreviations

DCC: *N*,*N*′-dicyclohexylcarbodiimide
DCM: dichloromethane
DIPEA: *N*,*N*-diisopropylethylamine
DLS: dynamic light scattering
DMAP: 4-dimethylaminopyridine
DMF: dimethylformamide
DMSO: dimethyl sulfoxide
DP: degree of polymerization
DPBS: Dulbecco’s phosphate-buffered saline
DTT: dithiothreitol
ECM: extracellular matrix
EtOAc: ethyl acetate
EVE: ethyl vinyl ether
FA: formic acid
Fmoc: fluorenylmethoxycarbonyl
FRET: Förster resonance energy transfer
G3: 3rd generation Grubbs catalyst
H&E: hematoxylin and eosin
HBTU: *N*,*N*,*N*′,*N*′-tetramethyl-*O*-(1*H*-benzotriazol-1-yl)uronium hexafluorophosphate
HCl: hydrochloric acid
Hex: hexane
HPLC: high-performance liquid chromatography
IR: ischemia-reperfusion
IV: intravenous
LV: left ventricle
MI: myocardial infarction
MMP: matrix metalloproteinase
MMPi: inhibitor of matrix metalloproteinase
MWCO: molecular-weight cutoff
NEt3: triethylamine
NHS: *N*-hydroxysuccinimide
NMR: nuclear magnetic resonance spectroscopy
NorHex: (*N*-hexanol)-5-norbornene-exo-2,3-dicarboximide
NorMMPi: PD166793 bearing norbornene monomer
NorNHS: *N*-hydroxysuccinimide-conjugated norbornene monomer
NorPh: phenyl norbornene monomer
NP: nanoparticle
PBS: phosphate-buffered saline
PPA: peptide-polymer amphiphile
PPM: post-polymerization modification
ROMP: ring-opening metathesis polymerization
SEC-MALS: size-exclusion chromatography coupled with multi-angle light scattering
SPPS: solid-phase peptide synthesis
TEM: transmission electron microscopy
TFA: trifluoroacetic acid
TIPS: triisopropyl silane
UV–Vis: ultraviolet–visible spectroscopy
ZDEC: zinc diethyldithiocarbamate
α-SMA: α-smooth muscle actin

## References

[ref1] Benjamin E. J.; Muntner P.; Alonso A.; Bittencourt M. S.; Callaway C. W.; Carson A. P.; Chamberlain A. M.; Chang A. R.; Cheng S.; Das S. R.; et al. Heart Disease and Stroke Statistics-2019 Update: A Report From the American Heart Association. Circulation 2019, 139, e56–e528. 10.1161/CIR.0000000000000659. 30700139

[ref2] Claesson-Welsh L. Vascular permeability—the essentials. Upsala J. Med. Sci. 2015, 120, 135–143. 10.3109/03009734.2015.1064501. 26220421 PMC4526869

[ref3] Roger V. L. Epidemiology of myocardial infarction. Med. Clin. North Am. 2007, 91, 537–552. 10.1016/j.mcna.2007.03.007. 17640535 PMC2537993

[ref4] Webb C. S.; Bonnema D. D.; Ahmed S. H.; Leonardi A. H.; McClure C. D.; Clark L. L.; Stroud R. E.; Corn W. C.; Finklea L.; Zile M. R.; et al. Specific temporal profile of matrix metalloproteinase release occurs in patients after myocardial infarction: relation to left ventricular remodeling. Circulation 2006, 114, 1020–1027. 10.1161/CIRCULATIONAHA.105.600353. 16923753

[ref5] Sutton M. G. S. J.; Sharpe N. Left Ventricular Remodeling After Myocardial Infarction. Circulation 2000, 101, 2981–2988. 10.1161/01.CIR.101.25.2981. 10869273

[ref6] French B. A.; Kramer C. M. Mechanisms of Post-Infarct Left Ventricular Remodeling. Drug Discov. Today Dis. Mech. 2007, 4, 185–196. 10.1016/j.ddmec.2007.12.006. 18690295 PMC2504336

[ref7] Vanhoutte D.; Schellings M.; Pinto Y.; Heymans S. Relevance of matrix metalloproteinases and their inhibitors after myocardial infarction: A temporal and spatial window. Cardiovasc. Res. 2006, 69, 604–613. 10.1016/j.cardiores.2005.10.002. 16360129

[ref8] Kaludercic N.; Lindsey M. L.; Tavazzi B.; Lazzarino G.; Paolocci N. Inhibiting metalloproteases with PD 166793 in heart failure: impact on cardiac remodeling and beyond. Cardiovasc. Drug Rev. 2008, 26, 24–37. 10.1111/j.1527-3466.2007.00034.x. 18466418

[ref9] Spinale F. G. Myocardial matrix remodeling and the matrix metalloproteinases: influence on cardiac form and function. Physiol. Rev. 2007, 87, 1285–1342. 10.1152/physrev.00012.2007. 17928585

[ref10] Yarbrough W. M.; Mukherjee R.; Escobar G. P.; Mingoia J. T.; Sample J. A.; Hendrick J. W.; Dowdy K. B.; McLean J. E.; Lowry A. S.; O’Neill T. P.; Spinale F. G. Selective targeting and timing of matrix metalloproteinase inhibition in post-myocardial infarction remodeling. Circulation 2003, 108, 1753–1759. 10.1161/01.CIR.0000091087.78630.79. 12975256

[ref11] Li K.; Tay F. R.; Yiu C. K. Y. The past, present and future perspectives of matrix metalloproteinase inhibitors. Pharmacol. Ther. 2020, 207, 107465 10.1016/j.pharmthera.2019.107465. 31863819

[ref12] Hudson M. P.; Armstrong P. W.; Ruzyllo W.; Brum J.; Cusmano L.; Krzeski P.; Lyon R.; Quinones M.; Theroux P.; Sydlowski D.; et al. Effects of selective matrix metalloproteinase inhibitor (PG-116800) to prevent ventricular remodeling after myocardial infarction: results of the PREMIER (Prevention of Myocardial Infarction Early Remodeling) trial. J. Am. Coll. Cardiol. 2006, 48, 15–20. 10.1016/j.jacc.2006.02.055. 16814643

[ref13] Purcell B. P.; Lobb D.; Charati M. B.; Dorsey S. M.; Wade R. J.; Zellars K. N.; Doviak H.; Pettaway S.; Logdon C. B.; Shuman J. A.; et al. Injectable and bioresponsive hydrogels for on-demand matrix metalloproteinase inhibition. Nat. Mater. 2014, 13, 653–661. 10.1038/nmat3922. 24681647 PMC4031269

[ref14] Fan Z.; Fu M.; Xu Z.; Zhang B.; Li Z.; Li H.; Zhou X.; Liu X.; Duan Y.; Lin P. H.; et al. Sustained Release of a Peptide-Based Matrix Metalloproteinase-2 Inhibitor to Attenuate Adverse Cardiac Remodeling and Improve Cardiac Function Following Myocardial Infarction. Biomacromolecules 2017, 18, 2820–2829. 10.1021/acs.biomac.7b00760. 28731675 PMC5723129

[ref15] Birnbaum Y.; Fishbein M. C.; Blanche C.; Siegel R. J. Ventricular Septal Rupture after Acute Myocardial Infarction. N. Engl. J. Med. 2002, 347, 1426–1432. 10.1056/NEJMra020228. 12409546

[ref16] Johnson T. D.; Christman K. L. Injectable hydrogel therapies and their delivery strategies for treating myocardial infarction. Expert Opin. Drug Del. 2013, 10, 59–72. 10.1517/17425247.2013.739156. 23140533

[ref17] Suarez S.; Almutairi A.; Christman K. L. Micro- and Nanoparticles for Treating Cardiovascular Disease. Biomater. Sci. 2015, 3, 564–580. 10.1039/C4BM00441H. 26146548 PMC4486363

[ref18] Ho Y. T.; Poinard B.; Kah J. C. Nanoparticle drug delivery systems and their use in cardiac tissue therapy. Nanomedicine 2016, 11, 693–714. 10.2217/nnm.16.6. 27003586

[ref19] Sullivan H. L.; Gianneschi N. C.; Christman K. L. Targeted nanoscale therapeutics for myocardial infarction. Biomater. Sci. 2021, 9, 1204–1216. 10.1039/D0BM01677B. 33367371 PMC7932032

[ref20] Dvir T.; Bauer M.; Schroeder A.; Tsui J. H.; Anderson D. G.; Langer R.; Liao R.; Kohane D. S. Nanoparticles targeting the infarcted heart. Nano Lett. 2011, 11, 4411–4414. 10.1021/nl2025882. 21899318 PMC3192253

[ref21] Xue X.; Shi X.; Dong H.; You S.; Cao H.; Wang K.; Wen Y.; Shi D.; He B.; Li Y. Delivery of microRNA-1 inhibitor by dendrimer-based nanovector: An early targeting therapy for myocardial infarction in mice. Nanomedicine 2018, 14, 619–631. 10.1016/j.nano.2017.12.004. 29269324

[ref22] Scott R. C.; Rosano J. M.; Ivanov Z.; Wang B.; Chong P. L.; Issekutz A. C.; Crabbe D. L.; Kiani M. F. Targeting VEGF-encapsulated immunoliposomes to MI heart improves vascularity and cardiac function. FASEB J. 2009, 23, 3361–3367. 10.1096/fj.08-127373. 19535683

[ref23] Zhang S.; Li J.; Hu S.; Wu F.; Zhang X. Triphenylphosphonium and D-α-tocopheryl polyethylene glycol 1000 succinate-modified, tanshinone IIA-loaded lipid-polymeric nanocarriers for the targeted therapy of myocardial infarction. Int. J. Nanomed. 2018, 13, 4045–4057. 10.2147/IJN.S165590. PMC6045899 30022826

[ref24] Zhang C. X.; Cheng Y.; Liu D. Z.; Liu M.; Cui H.; Zhang B. L.; Mei Q. B.; Zhou S. Y. Mitochondria-targeted cyclosporin A delivery system to treat myocardial ischemia reperfusion injury of rats. J. Nanobiotechnol. 2019, 17, 18 10.1186/s12951-019-0451-9. PMC6346555 30683110

[ref25] Monopoli M. P.; Åberg C.; Salvati A.; Dawson K. A. Biomolecular coronas provide the biological identity of nanosized materials. Nat. Nanotechnol. 2012, 7, 779 10.1038/nnano.2012.207. 23212421

[ref26] Nguyen M. M.; Carlini A. S.; Chien M. P.; Sonnenberg S.; Luo C.; Braden R. L.; Osborn K. G.; Li Y.; Gianneschi N. C.; Christman K. L. Enzyme-Responsive Nanoparticles for Targeted Accumulation and Prolonged Retention in Heart Tissue after Myocardial Infarction. Adv. Mater. 2015, 27, 5547–5552. 10.1002/adma.201502003. 26305446 PMC4699559

[ref27] Chien M. P.; Thompson M. P.; Barback C. V.; Ku T. H.; Hall D. J.; Gianneschi N. C. Enzyme-directed assembly of a nanoparticle probe in tumor tissue. Adv. Mater. 2013, 25, 3599–3604. 10.1002/adma.201300823. 23712821 PMC4108424

[ref28] Chien M. P.; Thompson M. P.; Lin E. C.; Gianneschi N. C. Fluorogenic Enzyme-Responsive Micellar Nanoparticles. Chem. Sci. 2012, 3, 2690–2694. 10.1039/c2sc20165h. 23585924 PMC3622269

[ref29] Spinale F. G.; Coker M. L.; Krombach S. R.; Mukherjee R.; Hallak H.; Houck W. V.; Clair M. J.; Kribbs S. B.; Johnson L. L.; Peterson J. T.; et al. Matrix metalloproteinase inhibition during the development of congestive heart failure: effects on left ventricular dimensions and function. Circ. Res. 1999, 85, 364–376. 10.1161/01.RES.85.4.364. 10455065

[ref30] Mukherjee R.; Brinsa T. A.; Dowdy K. B.; Scott A. A.; Baskin J. M.; Deschamps A. M.; Lowry A. S.; Escobar G. P.; Lucas D. G.; Yarbrough W. M.; et al. Myocardial infarct expansion and matrix metalloproteinase inhibition. Circulation 2003, 107, 618–625. 10.1161/01.CIR.0000046449.36178.00. 12566376

[ref31] Thompson M. P.; Randolph L. M.; James C. R.; Davalos A. N.; Hahn M. E.; Gianneschi N. C. Labelling polymers and micellar nanoparticles via initiation, propagation and termination with ROMP. Polym. Chem. 2014, 5, 1954–1964. 10.1039/C3PY01338C. 24855496 PMC4023353

[ref32] O’Brien P. M.; Ortwine D. F.; Pavlovsky A. G.; Picard J. A.; Sliskovic D. R.; Roth B. D.; Dyer R. D.; Johnson L. L.; Man C. F.; Hallak H. Structure-activity relationships and pharmacokinetic analysis for a series of potent, systemically available biphenylsulfonamide matrix metalloproteinase inhibitors. J. Med. Chem. 2000, 43, 156–166. 10.1021/jm9903141. 10649971

[ref33] Peterson J. T.; Hallak H.; Johnson L.; Li H.; O’Brien P. M.; Sliskovic D. R.; Bocan T. M. A.; Coker M. L.; Etoh T.; Spinale F. G. Matrix metalloproteinase inhibition attenuates left ventricular remodeling and dysfunction in a rat model of progressive heart failure. Circulation 2001, 103, 2303–2309. 10.1161/01.CIR.103.18.2303. 11342481

[ref34] Hazra S.; Guha R.; Jongkey G.; Palui H.; Mishra A.; Vemuganti G. K.; Basak S. K.; Mandal T. K.; Konar A. Modulation of matrix metalloproteinase activity by EDTA prevents posterior capsular opacification. Mol. Vis. 2012, 18, 1701–1711. 22815623 PMC3398497

[ref35] Singelyn J. M.; Sundaramurthy P.; Johnson T. D.; Schup-Magoffin P. J.; Hu D. P.; Faulk D. M.; Wang J.; Mayle K. M.; Bartels K.; Salvatore M.; et al. Catheter-deliverable hydrogel derived from decellularized ventricular extracellular matrix increases endogenous cardiomyocytes and preserves cardiac function post-myocardial infarction. J. Am. Coll. Cardiol. 2012, 59, 751–763. 10.1016/j.jacc.2011.10.888. 22340268 PMC3285410

[ref36] Dong H.; Pang L.; Cong H.; Shen Y.; Yu B. Application and design of esterase-responsive nanoparticles for cancer therapy. Drug Delivery 2019, 26, 416–432. 10.1080/10717544.2019.1588424. 30929527 PMC6450553

[ref37] DeLeon-Pennell K. Y.; Meschiari C. A.; Jung M.; Lindsey M. L. Matrix Metalloproteinases in Myocardial Infarction and Heart Failure. Prog. Mol. Biol. Transl. Sci. 2017, 147, 75–100. 10.1016/bs.pmbts.2017.02.001. 28413032 PMC5576003

[ref38] Lee H. B.; Blaufox M. D. Blood volume in the rat. J. Nucl. Med. 1985, 26, 72–76. 3965655

[ref39] Park J. C.; Park B. J.; Lee D. H.; Suh H.; Kim D. G.; Kwon O. H. Evaluation of the cytotoxicity of polyetherurethane (PU) film containing zinc diethyldithiocarbamate (ZDEC) on various cell lines. Yonsei. Med. J. 2002, 43, 518–526. 10.3349/ymj.2002.43.4.518. 12205741

[ref40] Callmann C. E.; Barback C. V.; Thompson M. P.; Hall D. J.; Mattrey R. F.; Gianneschi N. C. Therapeutic Enzyme-Responsive Nanoparticles for Targeted Delivery and Accumulation in Tumors. Adv. Mater. 2015, 27, 4611–4615. 10.1002/adma.201501803. 26178920 PMC4699560

[ref41] Battistella C.; Callmann C. E.; Thompson M. P.; Yao S. Y.; Yeldandi A. V.; Hayashi T.; Carson D. A.; Gianneschi N. C. Delivery of Immunotherapeutic Nanoparticles to Tumors via Enzyme-Directed Assembly. Adv. Healthcare Mater. 2019, 8, 1901105 10.1002/adhm.201901105. 31664791

[ref42] Battistella C.; Liang Y. F.; Gianneschi N. C. Innovations in Disease State Responsive Soft Materials for Targeting Extracellular Stimuli Associated with Cancer, Cardiovascular Disease, Diabetes, and Beyond. Adv. Mater. 2021, 33, 2007504 10.1002/adma.202007504. PMC9836048 34145625

[ref43] Callmann C. E.; Thompson M. P.; Gianneschi N. C. Poly(peptide): Synthesis, Structure, and Function of Peptide-Polymer Amphiphiles and Protein-like Polymers. Acc. Chem. Res. 2020, 53, 400–413. 10.1021/acs.accounts.9b00518. 31967781 PMC11042489

