# Supplementary material for: Enzyme-Responsive Nanoparticles for the Targeted Delivery of an MMP Inhibitor to Acute Myocardial Infarction
Source: Biomacromolecules. 2023 Sep 11;24(11):4695–704. doi: 10.1021/acs.biomac.3c00421 (PMC10646957; doi:10.1021/acs.biomac.3c00421)
Supplement: Supplementary file 1 — bm3c00421_si_001.pdf [file bm3c00421_si_001.pdf]

Supporting Information

Enzyme-Responsive Nanoparticles for the Targeted Delivery of an MMP Inhibitor to the Heart post Myocardial Infarction

Holly L. Sullivan,‡[1] Yifei Liang,‡[2] Kendra Worthington,[1] Colin Luo,[1] Nathan C. Gianneschi,[2,3,4] Karen L. Christman,*[1]*

[1]Shu Chien-Gene Lay Department of Bioengineering and the Sanford Consortium for Regenerative, Medicine, University of California San Diego, La Jolla, USA
[2]Department of Chemistry, International Institute for Nanotechnology, Simpson-Querrey Institute, Chemistry of Life Processes Institute, Northwestern University, Evanston, Illinois 60208, United States.
[3]Departments of Materials Science & Engineering, Biomedical Engineering and Pharmacology, Northwestern University, Evanston, Illinois 60208, United States.
[4]Department of Chemistry & Biochemistry, University of California San Diego, La Jolla, United States

‡These authors contributed equally to this work.
AUTHORS EMAIL ADDRESS:

h1sulliv@eng.ucsd.edu,

yifeiliang2022@u.northwestern.edu,

klworthi@ucsd.edu,

cluo@eng.ucsd.edu,

christman@eng.ucsd.edu,

nathan.gianneschi@northwestern.edu.

CORRESPONDING AUTHOR FOOTNOTE:

nathan.gianneschi@northwestern.edu, christman@eng.ucsd.edu

Table of Contents

1. Supporting Tables 3
2. Supporting Figures 5
3. Supporting Spectra 10
4. Reference 18

1. Supporting Tables

Table S1. SEC-MALS characterization of original PPA and intermediates.

	**$NorPh_{13}$-*co*-$NorMMPi_5$**	**Intermediate block copolymer**	**Original PPA**
$M_{n,\ theo}$ **(kDa)** [a]	7.9	9.1	16.2
$M_{n,\ MALS}$ **(kDa)** [b]	7.0	8.0	13.0
Đ	1.01	1.01	1.02

[a]Theoretical molecular weight $M_{n,theo} = \Sigma\ DP_{monomer} \times MW_{monomer}$. [b]Molecular weight and dispersity were determined by SEC-MALS with a *dn/dc* of 0.179 mL/g in DMF with 0.05 M LiBr.

Table S2. SEC-MALS characterization of **PPA_{Max}**, **PPA_C** and intermediates.

	**$NorMMPi_{20}$**	**$NorMMPi_{20}$-*b*-$NorNHS_5$**	**PPA_{Max}**	**$NorPh_{20}$**	**$NorPh_{20}$-*b*-$NorNHS_5$**	**PPA_C**
$M_{n,\ theo}$ **(kDa)** [a]	13.2	14.3	20.9	5.1	6.2	13.4
$M_{n,\ MALS}$ **(kDa)** [b]	9.0	10.0	16.0	5.5	6.0	13.5
Đ	1.01	1.01	1.06	1.02	1.01	1.15

[a]Theoretical molecular weight $M_{n,theo} = \sum DP_{monomer} \times MW_{monomer}$. [b]Molecular weight and dispersity were determined by SEC-MALS with a *dn/dc* of 0.179 mL/g in DMF with 0.05 M LiBr.

Table S3. Summary of PPA drug dosage.

	**Original PPA**	**PPA_{Max}**
**NorPh: NorMMPi (m:n)**	13:7	0:20
**Polymer MW (g/mol)**	13360	20890
**Drug weight (g/mol polymer)**	2890	8240
**Drug mass (mg/300 nmol polymer)**	0.87	2.5
**Drug moles (μmole/300 nmol polymer)**	2.1	6.0
**Drug wt%**	20%	40%
**Drug loading (mg/Kg)**	3.5	10
**Drug loading (μmol/L)**	124	353

2. Supporting Figures

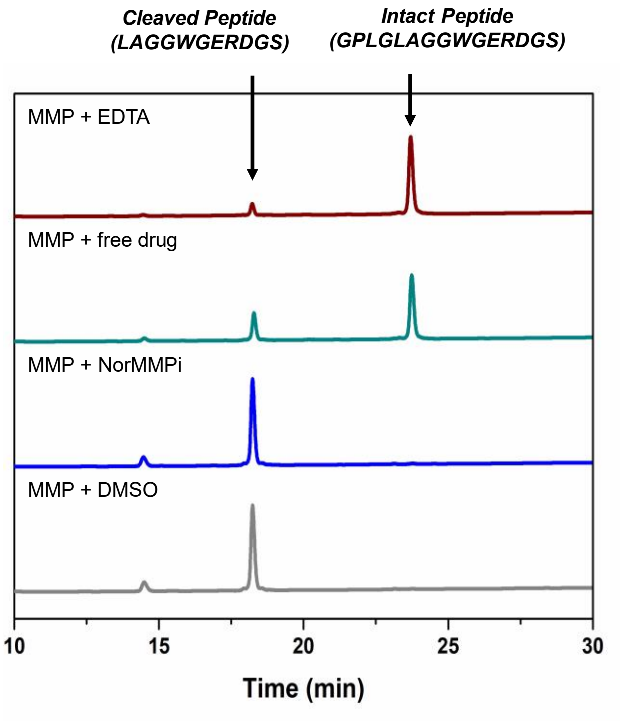

Figure S1. HPLC-UV analysis of MMP cleavable peptide post incubation with MMP-9 treated with DMSO, NorMMPi, free PD166793 or EDTA.

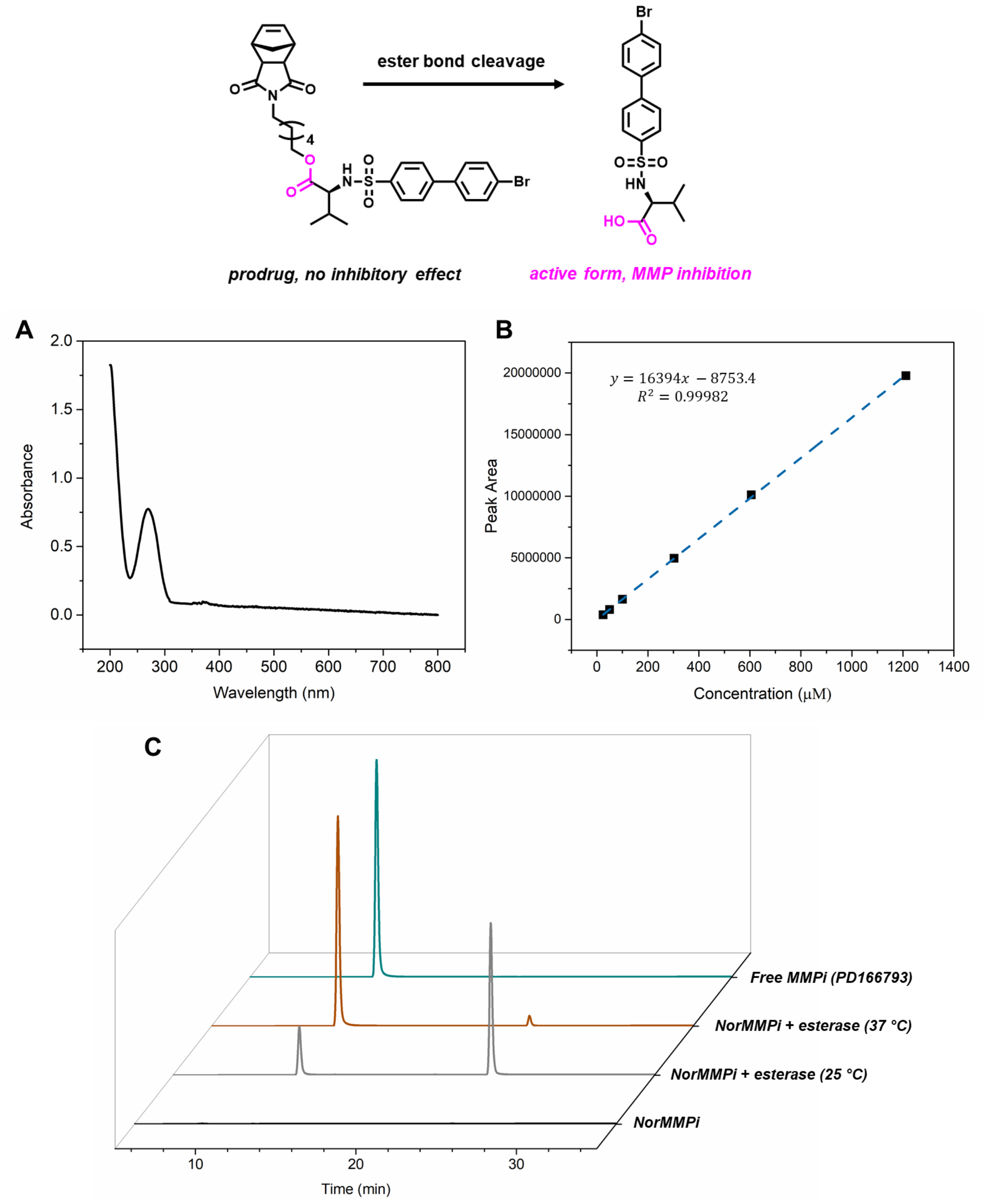

Figure S2. Esterase-catalyzed PD166793 release from NorMMPi. (A) UV-Vis spectrum of PD166793 in acetonitrile. Maximum absorbance was detected at 270 nm. (B) Calibration curve of PD166793 by HPLC-UV at 270 nm. (C) HPLC traces of NorMMPi alone, NorMMPi post 24 h esterase treatment at 25 and 37 °C, and PD166793 control.

By integrating free MMPi peak area, 20% MMPi was released from NorMMPi at 25 °C and 90% MMPi was released at 37 °C.

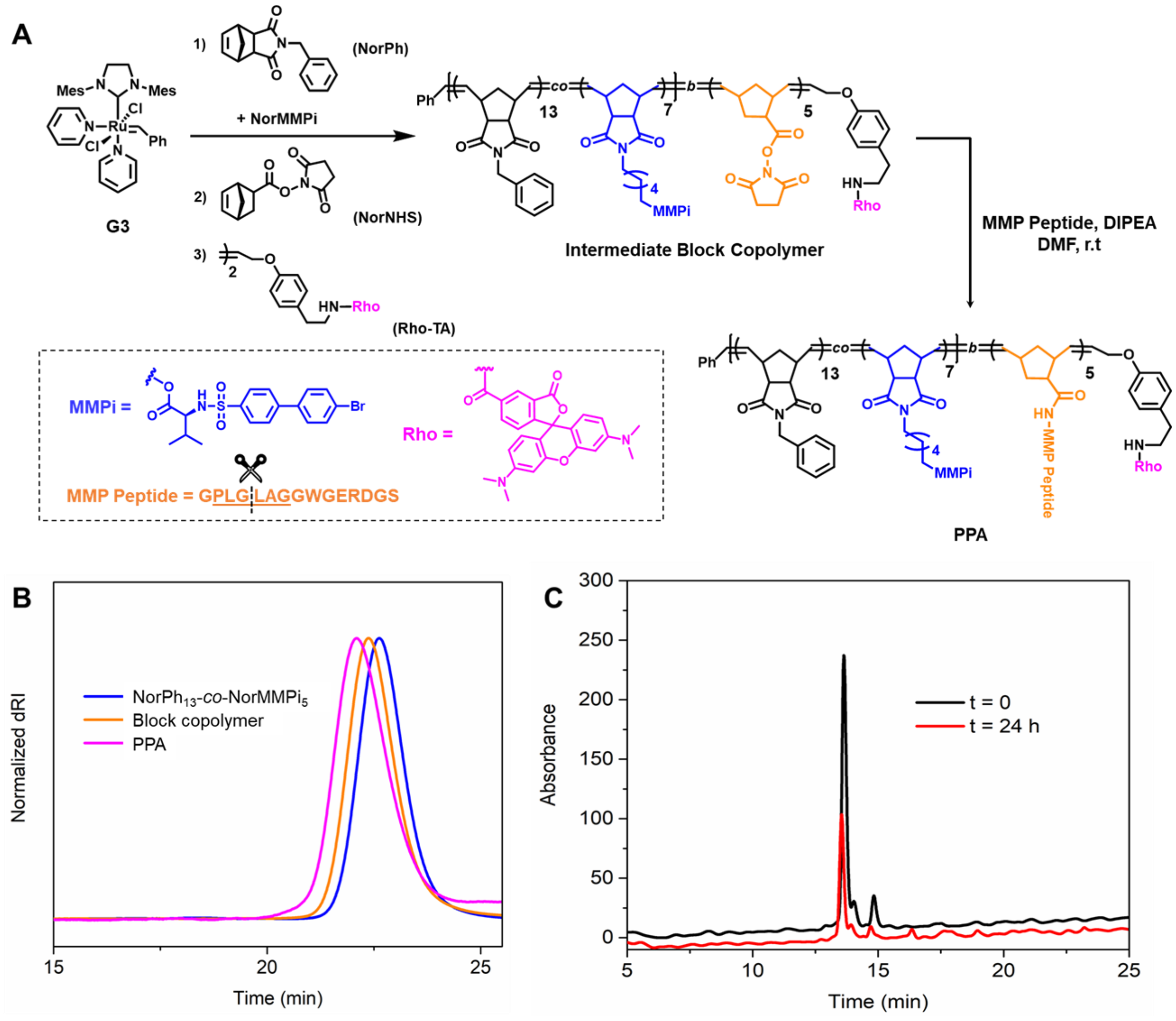

Figure S3. Synthesis of PD166793-incorporated peptide-polymer amphiphile (PPA). (A) Reaction scheme for PPA preparation. (B) SEC traces of $NorPh_{13}$-*co*-$NorMMPi_5$, the intermediate block copolymer and PPA post PPM. Polymer molecular weights and dispersities are summarized in **Table S1**. (C) MMP peptide consumption monitored by HPLC at 214 nm.

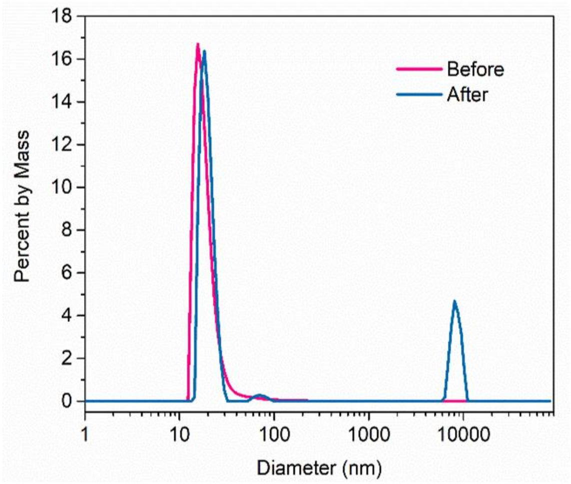

Figure S4. DLS analysis of PD166793 loaded nanoparticles (NPs) before and after thermolysin treatment.

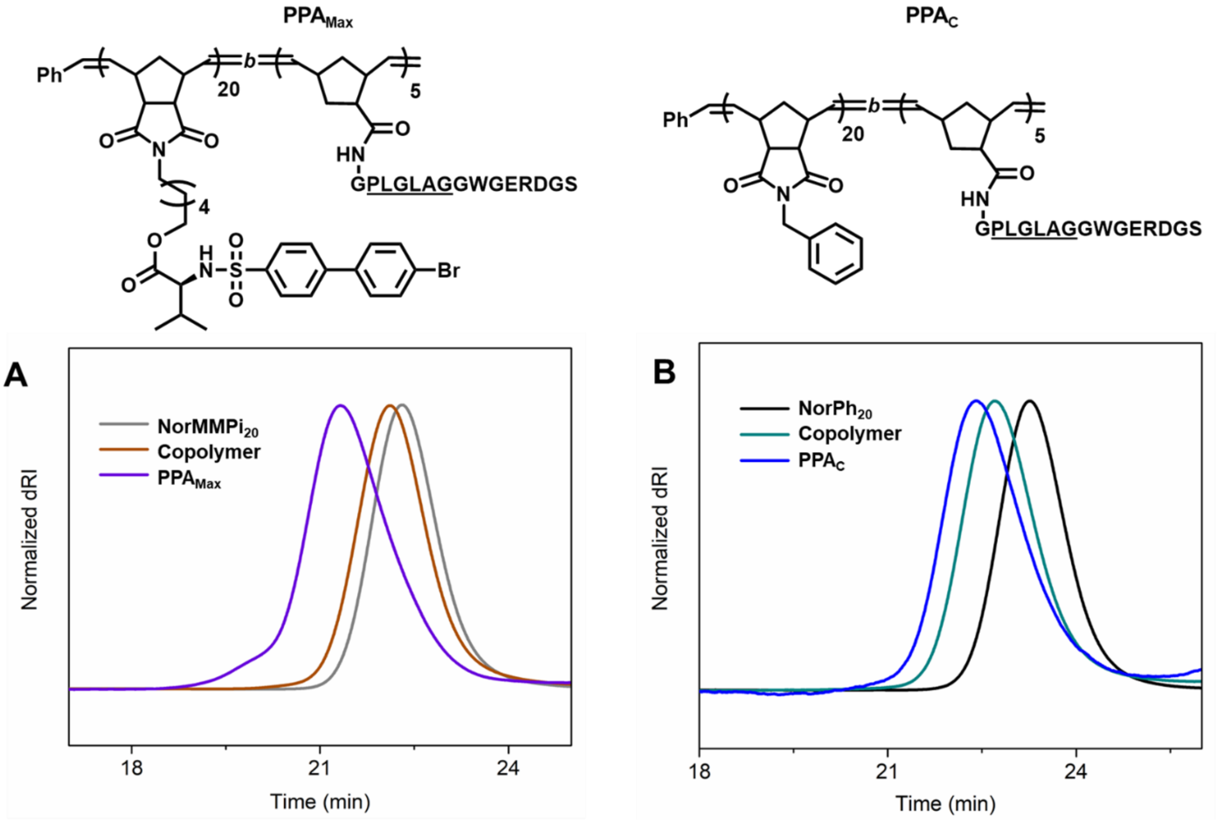

Figure S5. Synthesis of **PPA$_{Max}$** and **PPA$_C$**. (A) SEC traces of NorMMPi$_{20}$, the intermediate copolymer (NorMMPi$_{20}$-*b*-NorNHS$_5$) and **PPA$_{Max}$.** (B) SEC traces of NorPh$_{20}$, the intermediate copolymer (NorPh$_{20}$-*b*-NorNHS$_5$) and **PPA$_C$**. Polymer molecular weights and dispersities are summarized in **Table S2**.

3. Supporting Spectra

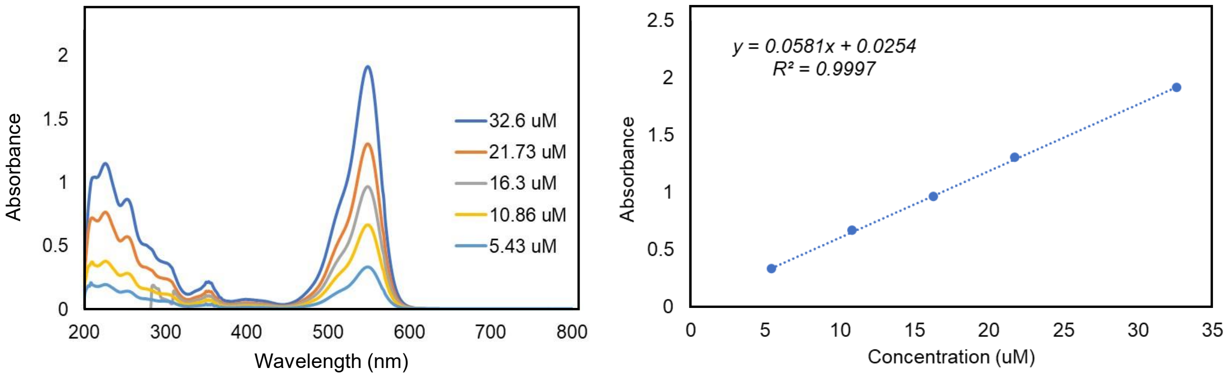

Figure S6. UV absorbance of Rho-TA (left) and calibration curve at 548 nm (right).

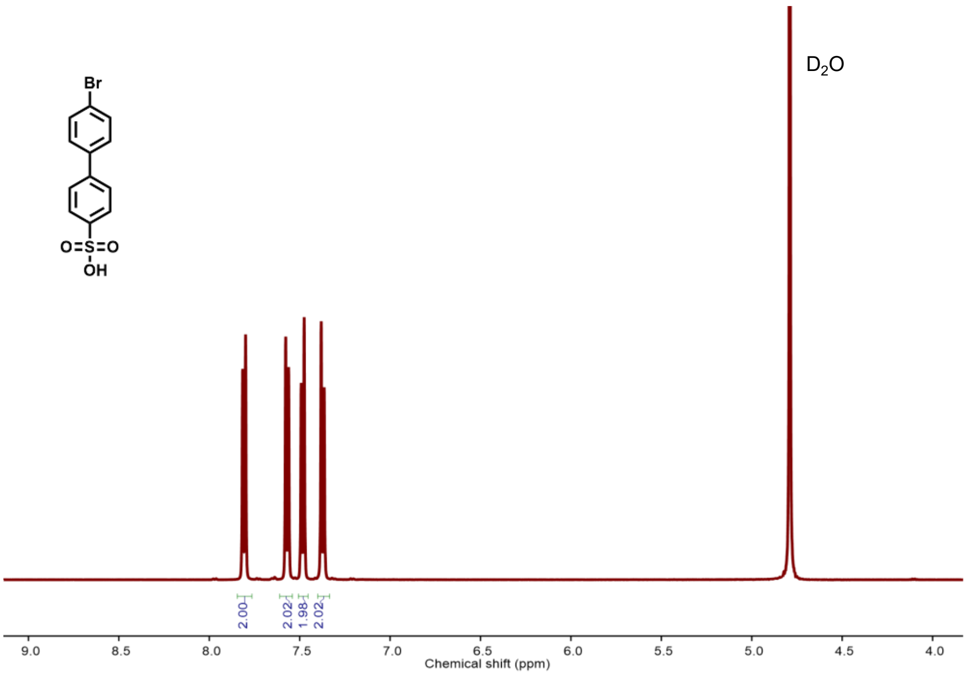

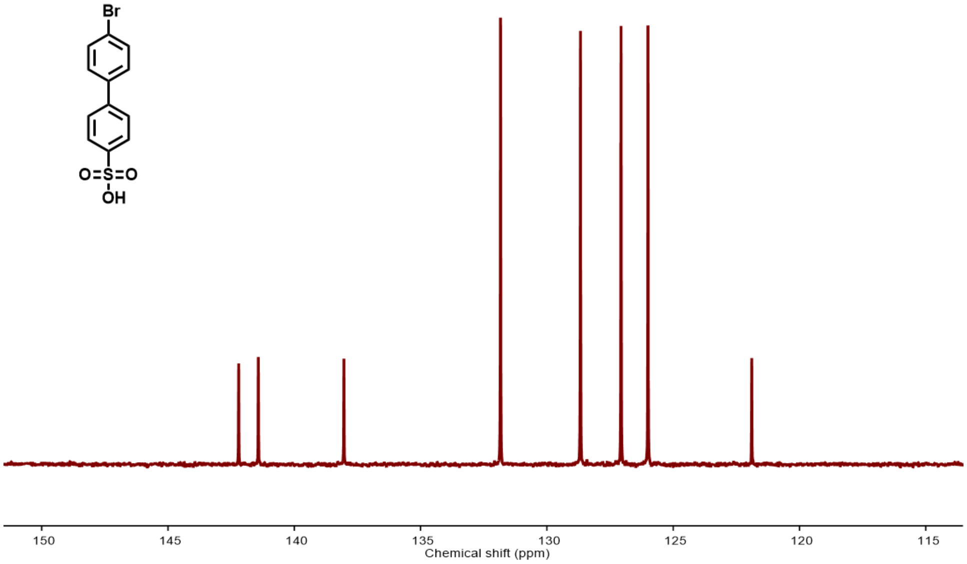

Figure S7. ^{1}H (top) and ^{13}C NMR (bottom) spectra of 4'-bromobiphenyl-4-sulfonic acid in D_2O.

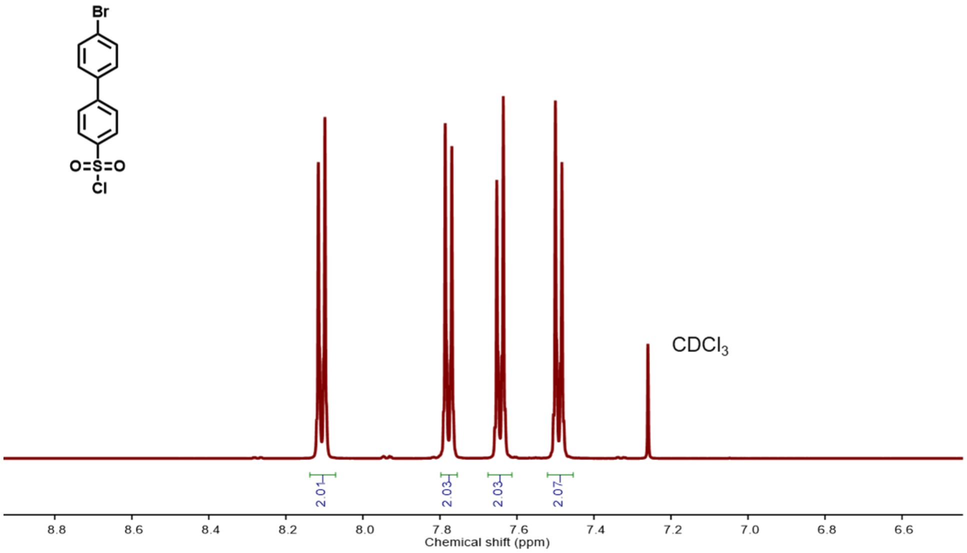

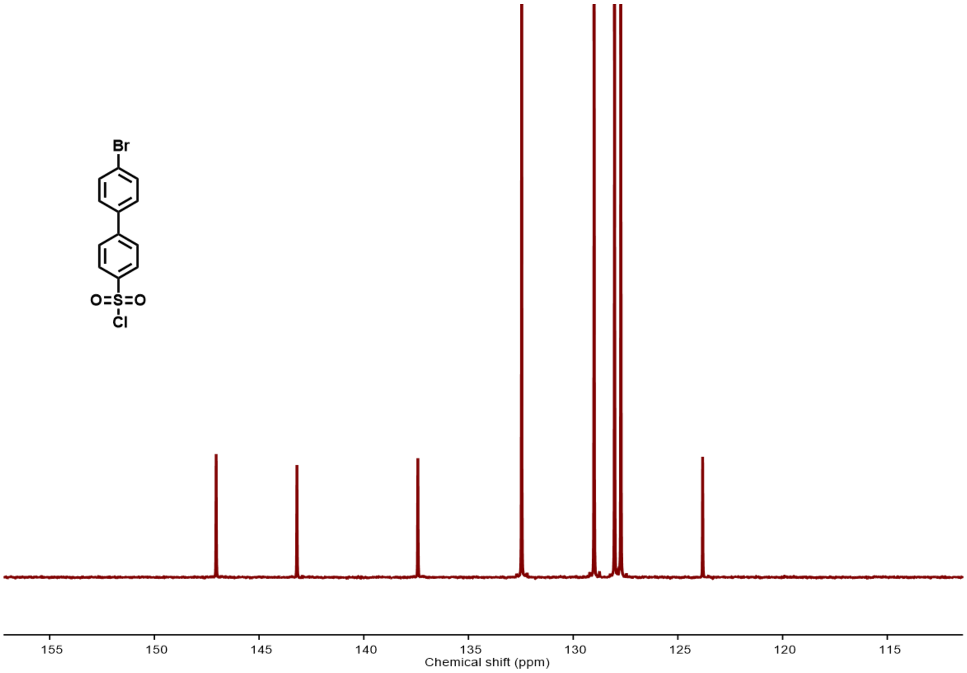

Figure S8. ^{1}H (top) and ^{13}C NMR (bottom) spectra of 4'-bromobiphenyl-4-sulfonyl chloride in $CDCl_3$.

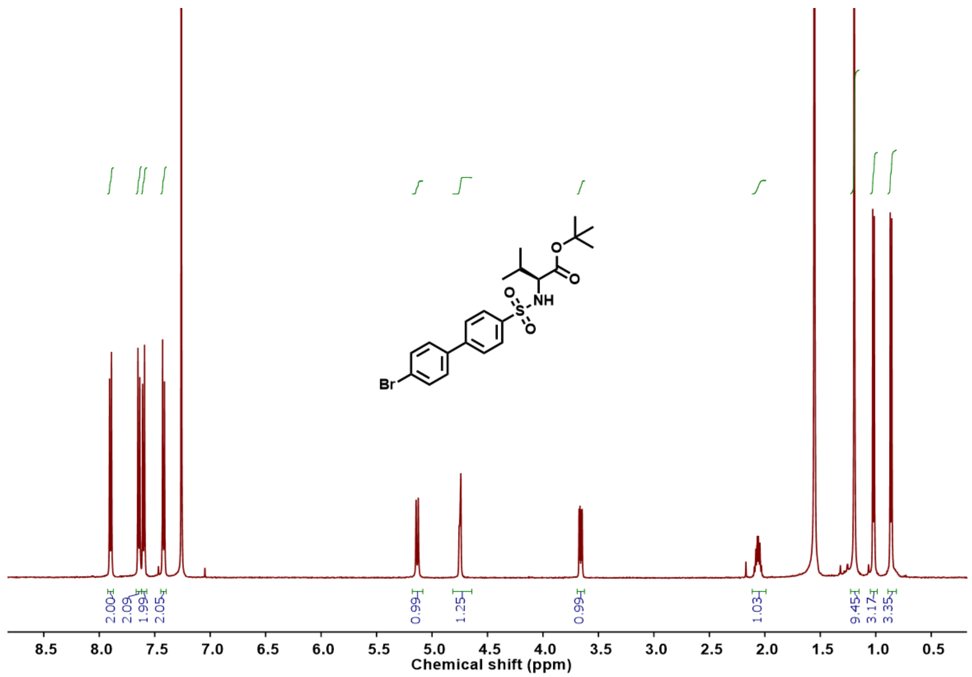

Figure S9. ^{1}H NMR spectrum of t-butyl ester protected PD166793 in $CDCl_3$.

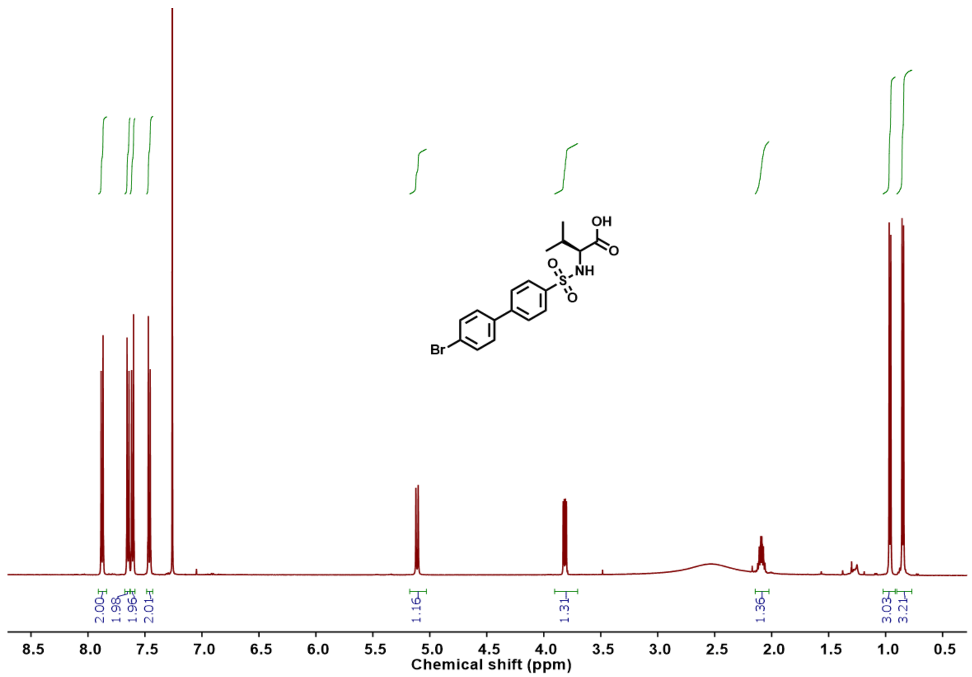

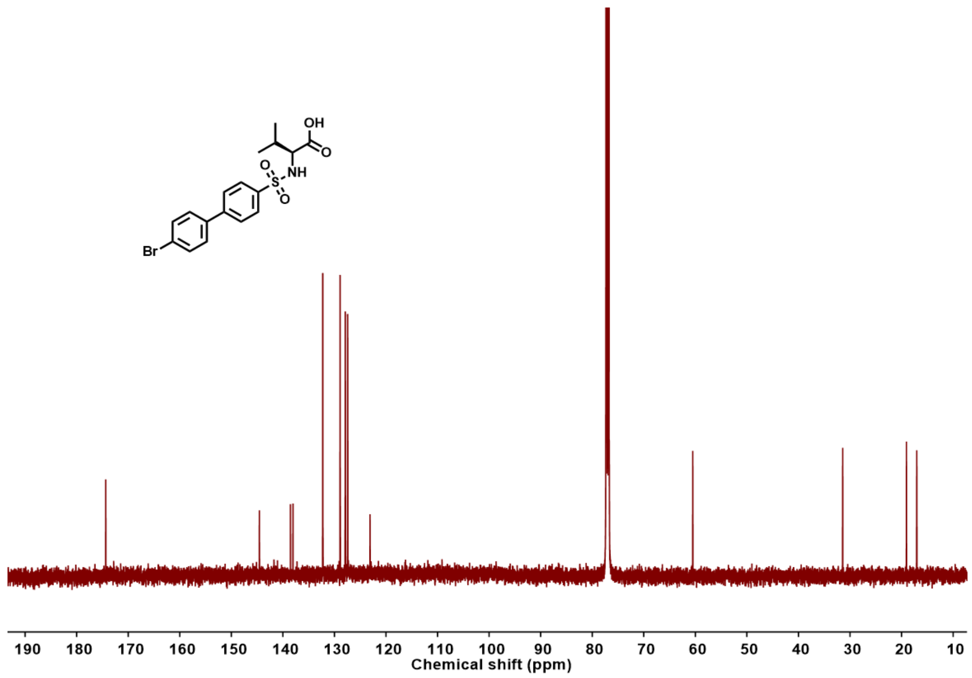

Figure S10. ^{1}H (top) and ^{13}C (bottom) NMR spectra of PD166793 in $CDCl_3$.

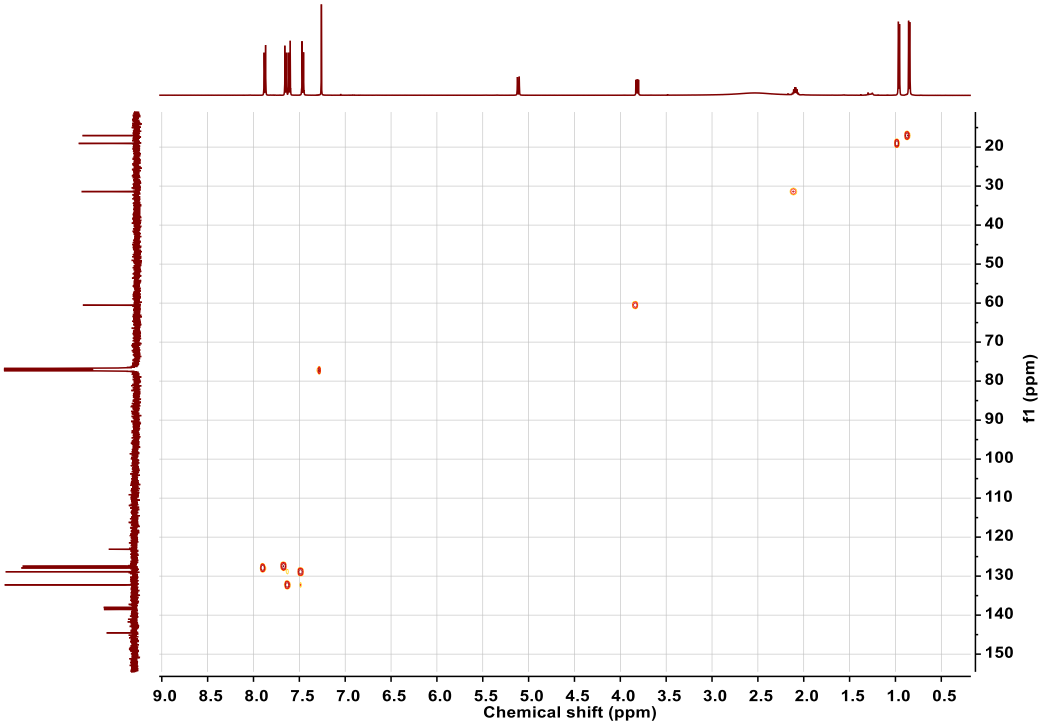

Figure S11. HSQC spectrum of PD166793 in $CDCl_3$.

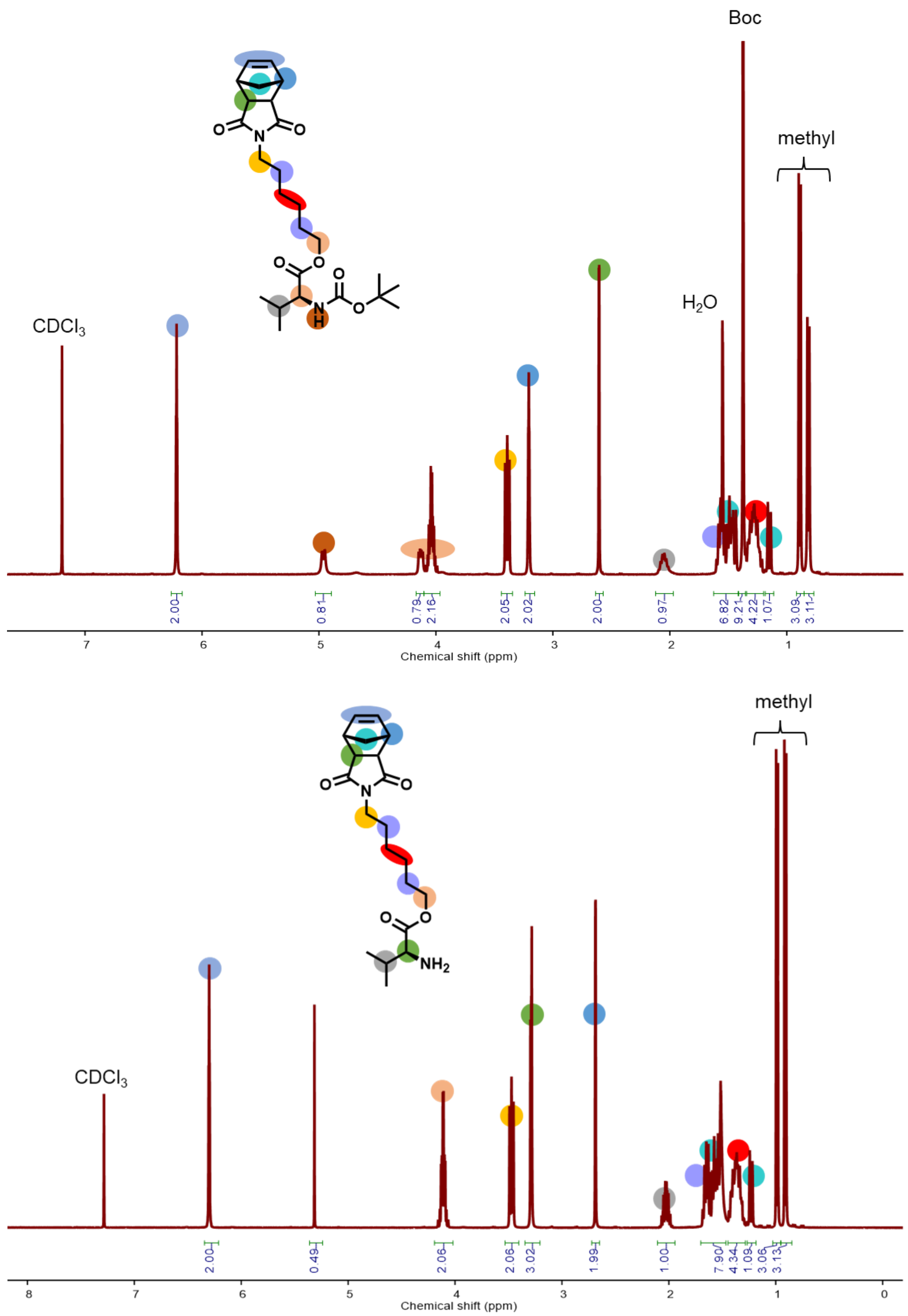

Figure S12. ^{1}H NMR spectra of NorHex-Boc-Valine (top) and NorHex-Valine (bottom) in $CDCl_3$.

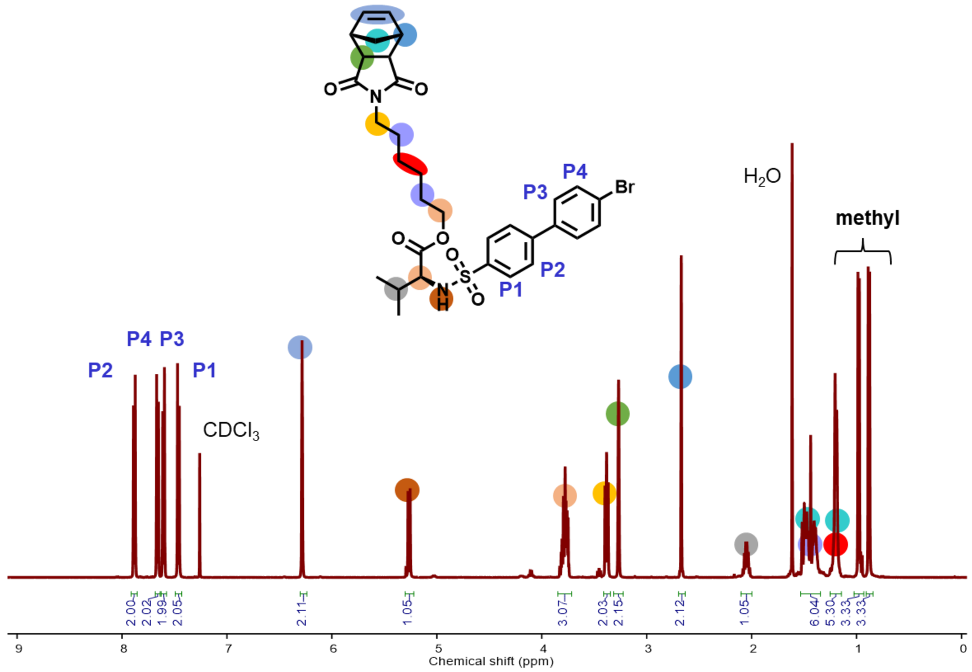

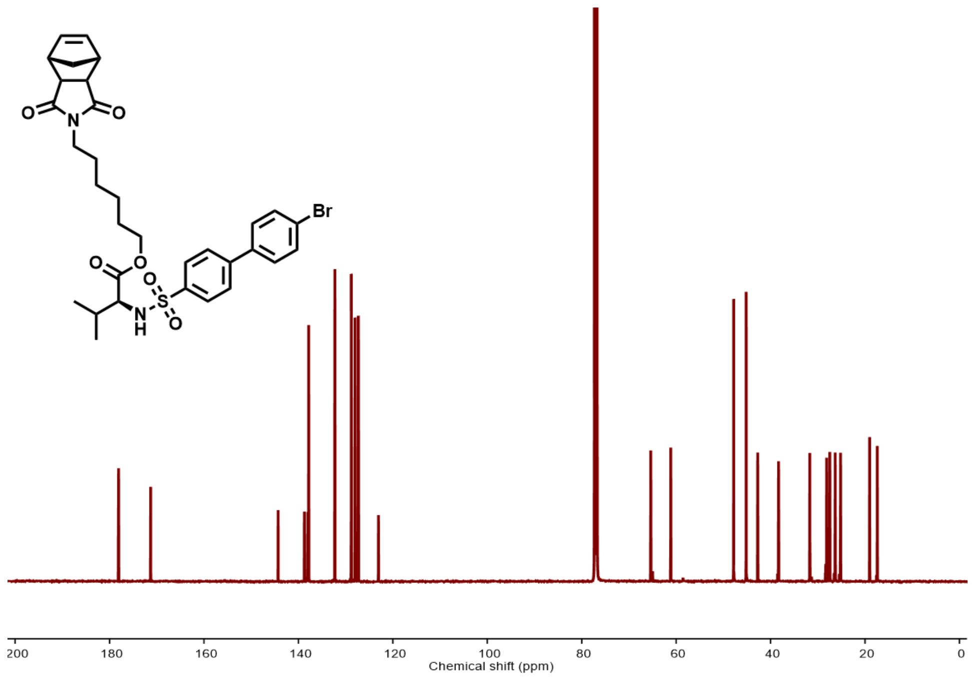

Figure S13. ^{1}H (top) and ^{13}C (bottom) NMR spectra of NorMMPi in $CDCl_3$.

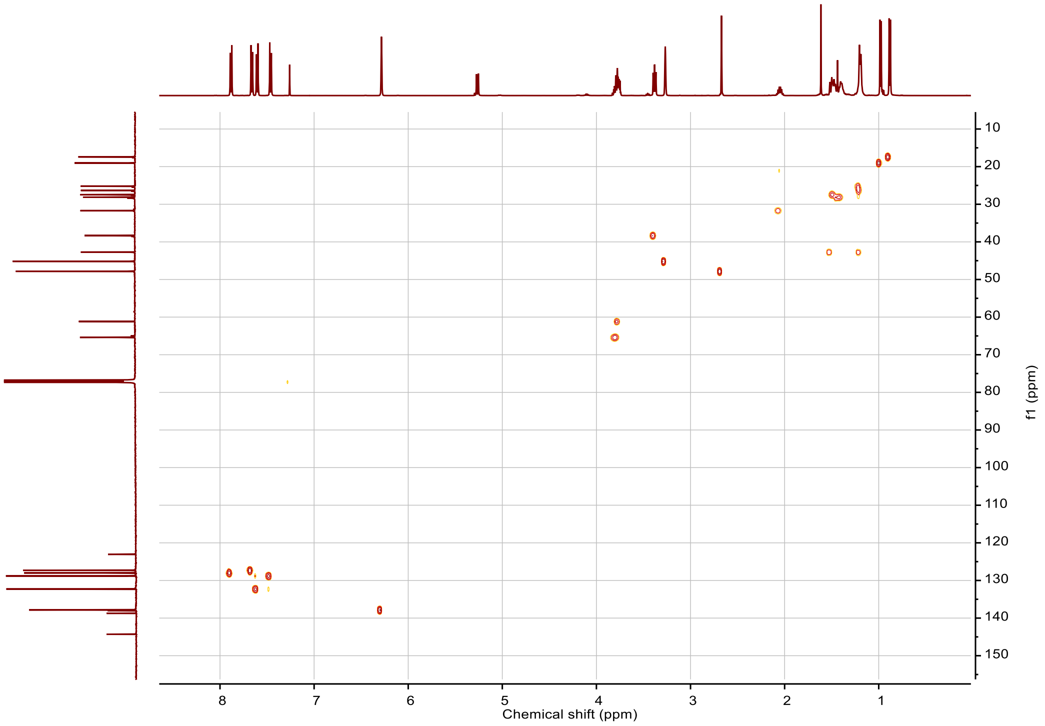

Figure S14. HSQC spectrum of NorMMPi in $CDCl_3$.

4. Reference

1. Thompson, M.P. et al. Labelling polymers and micellar nanoparticles via initiation, propagation and termination with ROMP. *Polym Chem-Uk* **5**, 1954-1964 (2014).
2. Nguyen, M.M. et al. Enzyme-Responsive Nanoparticles for Targeted Accumulation and Prolonged Retention in Heart Tissue after Myocardial Infarction. *Adv Mater* **27**, 5547-5552 (2015).
3. Peterson, J.T. et al. Matrix metalloproteinase inhibition attenuates left ventricular remodeling and dysfunction in a rat model of progressive heart failure. *Circulation* **103**, 2303-2309 (2001).
4. Hazra, S. et al. Modulation of matrix metalloproteinase activity by EDTA prevents posterior capsular opacification. *Mol. Vis.* **18**, 1701-1711 (2012).
5. Singelyn, J.M. et al. Catheter-deliverable hydrogel derived from decellularized ventricular extracellular matrix increases endogenous cardiomyocytes and preserves cardiac function post-myocardial infarction. *J. Am. Coll. Cardiol.* **59**, 751-763 (2012).
